# Bioactive plant and fungal metabolites in oral cancer: molecular mechanisms and translational potential

**DOI:** 10.3389/fphar.2026.1794285

**Published:** 2026-04-01

**Authors:** Yi Luo, Xijie Wu, Wenjing Chen, Ziao He, Yan Yang, Linjie Ma, Hongqiang Chen, Sicheng Zhang, Chao Li

**Affiliations:** 1 Department of Head and Neck Surgery, Sichuan Clinical Research Center for Cancer, Sichuan Cancer Hospital and Institute, Sichuan Cancer Center, University of Electronic Science and Technology of China, Chengdu, China; 2 School of Clinical Medicine, Chengdu University of Traditional Chinese Medicine, Chengdu, China; 3 Chengdu Integrated TCM & Western Medicine Hospital, Chengdu University of Traditional Chinese Medicine Affiliated Hospital of Integrated Traditional Chinese and Western Medicine, Chengdu, China

**Keywords:** bioactive metabolites, clinical transformation, molecular mechanisms, natural products, oral cancer, traditional Chinese medicine

## Abstract

Oral cancer remains a major global health burden. Current therapies are often limited by recurrence, drug resistance, and systemic toxicity. Natural bioactive metabolites, particularly those derived from medicinal plants used in Traditional Chinese Medicine are increasingly investigated as multitarget anticancer leads. In this review, we integrated evidence from studies published between January 2010 and June 2025. We summarize representative flavonoids, terpenoids, phenolic metabolites, alkaloids, polysaccharides, and quinones evaluated in oral cancer models. Reported activities include suppression of proliferation, induction of apoptosis and autophagy, inhibition of epithelial–mesenchymal transition and angiogenesis, and modulation of tumor metabolism and immune pathways. However, the current evidence base remains largely preclinical and frequently relies on simplified *in vitro* assays. Pharmacokinetic bridging, toxicity evaluation, and clinically relevant validation are still limited. Importantly, several polyphenolic metabolites exhibit pan-assay interference (PAINS) liabilities, requiring cautious interpretation of pathway-level findings. Advances in nano-delivery systems, rational structural optimization, and combination strategies may improve exposure and selectivity. Future work should emphasize PAINS-aware pharmacological rigor, orthogonal target validation, advanced translational models, and biomarker-driven clinical trials to support the development of genuinely druggable natural metabolite candidates for oral cancer management.

## 1 Introduction

**FIGURE 1:**
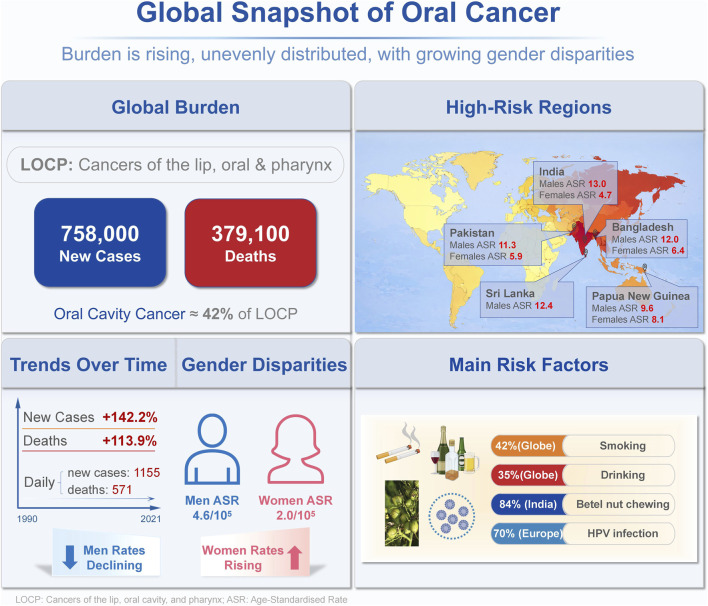
Global snapshot of oral cavity cancer burden, high-risk regions, gender disparities, and the main risk factors. This infographic summarises the global epidemiology of oral cavity cancer, as estimated by GLOBOCAN 2022. It highlights the overall incidence and mortality of cancers of the lip, oral cavity and pharynx (LOCP), with cancers of the oral cavity accounting for around 42% of LOCP cases. The map shows the countries with the highest incidence rates (India, Sri Lanka, Bangladesh, Pakistan and Papua New Guinea), which are presented as sex-specific age-standardised rates (ASR). Temporal trends from 1990 to 2021 show a significant rise in global incident cases and deaths, alongside ongoing gender disparities, with a higher ASR observed in men than in women. Key risk factors contributing to geographic variation are also illustrated.

Oral cancer remains a major global public health challenge. Its incidence continues to rise, with pronounced regional disparities and sex-specific trends (Figure 1) (Meng S. et al., 2025; Rumgay et al., 2026). The prognosis is often poor because many patients are diagnosed at advanced stages. In several Asian regions, the combination of betel nut chewing, tobacco use, and heavy alcohol consumption further increases the disease burden. (Sun et al., 2023). Epidemiological projections suggest that the incidence of oral cancer in these regions will remain high until around 2030 (Lu et al., 2023). Histologically, oral squamous cell carcinoma (OSCC) accounts for the vast majority of oral cancer cases. Less common subtypes include adenocarcinoma, basal cell carcinoma, undifferentiated carcinoma, and lymphoepithelial carcinoma. Multiple factors drive oral tumorigenesis, including genetic alterations, oncogenic viral infection, chronic irritation, and immune dysregulation (Menon et al., 2025). Current therapeutic approaches mainly focus on surgical resection, often combined with radiotherapy, chemotherapy, targeted therapy, and immunotherapy (Melo-Alvim et al., 2022). Additionally, neoadjuvant chemotherapy is also being explored to downsize tumors and improve resectability in selected patients (Siafa et al., 2025). Nevertheless, substantial challenges remain, including local invasion and recurrence, distant metastasis, heterogeneous therapeutic responses, primary or acquired drug resistance, and treatment-related toxicity (Khera et al., 2023; Kim et al., 2024; Noronha et al., 2024). These limitations underscore the urgent need for adjunctive, multitarget strategies that can complement standard care.

A significant proportion of oncology-relevant bioactive metabolites originate from natural products that have long been used in traditional medical systems, especially TCM. Additionally, all of the species or herbal medicines examined in this review are documented in national or regional pharmacopoeia monographs. TCM-derived metabolites (TCM monomers) refer to purified compounds isolated from botanical drugs with well-defined chemical structures and characterized biological activities. Extensive research has demonstrated that various TCM monomers possess multi-metabolite, multi-target, and multi-pathway antitumor properties across diverse cancer types (Wang S. et al., 2023). Reported effects include regulation of proliferation and apoptosis, suppression of epithelial–mesenchymal transition (EMT), inhibition of migration and invasion, metabolic reprogramming, anti-angiogenic activity, and modulation of the tumor immune microenvironment (Yang et al., 2025). In specific experimental or clinical conditions, certain monomers have been observed to demonstrate antitumor efficacy comparable to that of conventional chemotherapeutic agents. For instance, matrine, when formulated with nanocarriers, has demonstrated remarkable potential to reverse multidrug resistance in preclinical models, signifying its substantial promise for facilitating the transition from fundamental research to clinical application (Meng D. et al., 2025; Yang et al., 2025). These metabolites have been observed to exert synergistic or sensitizing effects when employed in conjunction with chemotherapy, radiotherapy, immune checkpoint inhibitors, or targeted therapies. For instance, ginsenosides (Xi et al., 2025) and curcumin (Wu et al., 2024) have demonstrated significant antitumor potential in various solid tumor models by inducing programmed cell death, regulating critical signaling pathways such as NF-κB, PI3K/Akt, and JAK/STAT, overcoming drug resistance, and regulating immune responses (Weng and Goel, 2022).

**FIGURE 2:**
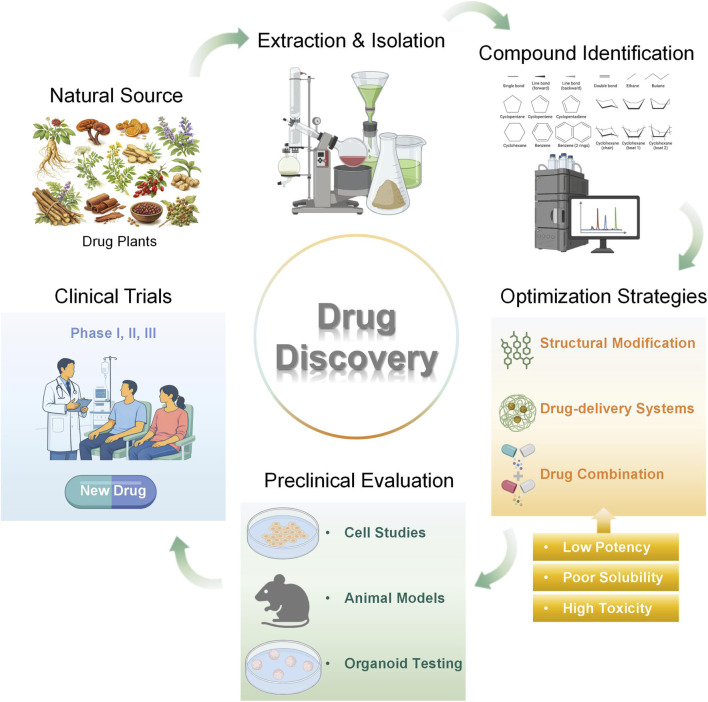
Translational pathway from natural products to clinical applications.

In the context of oral cancer specifically, there is considerable therapeutic potential in TCM-derived metabolites. Recent advancements in the fields of biotechnology and pharmacology have indicated that TCM exerts anticancer activity through the interplay of multiple targets and signaling pathways (Lu et al., 2021). A mounting body of evidence from *in vitro* and *in vivo* experiments suggests that multiple TCM monomers inhibit oral cancer progression through diverse molecular mechanisms. These mechanisms involve the suppression of cellular proliferation (Hu M. et al., 2023), the induction of cell-cycle arrest and apoptosis (Wang et al., 2017), the regulation of autophagy (Fukuda et al., 2022), the inhibition of EMT, invasion, and metastasis (Kim et al., 2020), the inhibition of angiogenesis (Yu et al., 2024), the attenuation of drug resistance (Chang et al., 2021), and the regulation of the immune system (Kawaguchi et al., 2022). Furthermore, these agents have demonstrated efficacy as monotherapies or in combination with radiotherapy and chemotherapy (Chen L. et al., 2020). Concurrently, the field of bionanotechnology has witnessed a marked acceleration in the optimization of formulations and the conduct of preliminary pharmacokinetic and toxicological assessments. In this review, we systematically summarize the molecular mechanisms of natural bioactive metabolites in oral cancer, with a particular focus on TCM-derived candidates. We further discuss evidence gaps, translational barriers, and future directions for clinical development (Figure 2).

## 2 Review methodology

**FIGURE 3:**
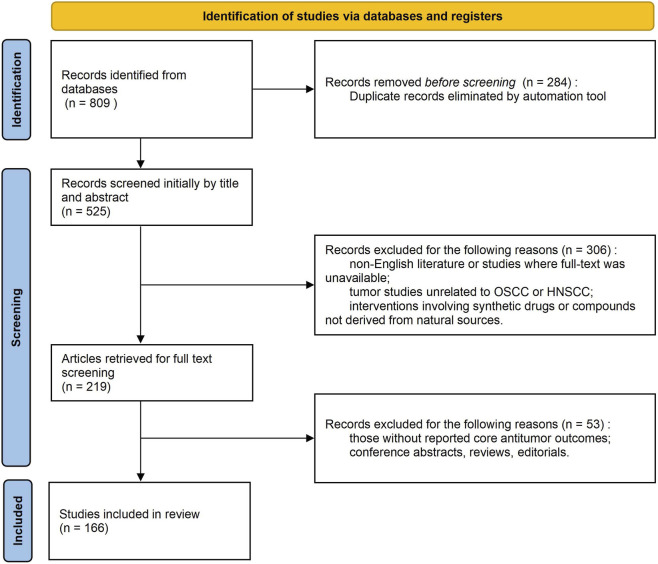
The literature search and screening flowchart.

This review systematically integrates literature on the antitumor effects of natural bioactive metabolites in oral cancer (Figure 3). Evidence from relevant studies published between January 2010 and June 2025 was included through searches of the PubMed, Web of Science, and Embase databases. The search terms encompassed keyword combinations such as “oral squamous cell carcinoma,” “head and neck squamous cell carcinoma,” “natural products,” “plant-derived metabolites,” “plant metabolites,” and “phytochemicals.” These terms were supplemented with those related to mechanisms and translation, including “apoptosis,” “autophagy,” “ferroptosis,” “tumor microenvironment,” and “nano-delivery system.” The inclusion criteria were: (i) studies that explicitly used oral cancer-related models, including oral cancer cell lines, animal xenograft models, or clinical samples; (ii) interventions that involved purified monomeric metabolites derived from natural products or structurally characterized plant metabolites (e.g., flavonoids, alkaloids, terpenoids, and polyphenols), with clearly defined metabolite names and sources; and (iii) study types that included *in vitro* experiments, *in vivo* animal studies, or preclinical mechanism research that reported explicit antitumor outcome measures. The exclusion criteria included the following: (i) publications in languages other than English; (ii) studies unrelated to OSCC/HNSCC tumors that did not conduct independent analyses; (iii) interventions involving synthetic drugs or metabolites not sourced naturally; (iv) studies failing to report core antitumor outcomes; and (v) conference abstracts, review articles, editorials, duplicate publications, or studies without full-text availability. Two researchers performed the initial title/abstract screening and full-text evaluation independently. Disagreements were resolved through discussion or third-party adjudication. Ultimately, 166 articles were included in the systematic analysis. The vast majority of these articles focused on the anti-proliferative, pro-apoptotic, anti-invasive/metastatic, and immune microenvironment-modulating effects of natural monomeric metabolites in OSCC models. These articles provided systematic evidence for mechanistic research and the clinical translation of natural products.

All botanical drug sources were taxonomically validated using Plants of the World Online (POWO, Kew), and scientific names are provided with authorities and family assignments.

## 3 Classification of bioactive metabolites derived from natural products

### 3.1 Flavonoids

Flavonoids display diverse pharmacological activities, including antioxidant, anti-inflammatory, anticancer, and immunomodulatory activities. In oral cancer, flavonoid monomers exert multifaceted anticancer actions through multiple signaling pathways. Reported mechanisms include suppression of cell proliferation with attendant cell-cycle arrest; induction of apoptosis and autophagy; inhibition of EMT, invasion, and metastasis; blockade of angiogenesis; reprogramming of tumor and cellular metabolism; modulation of the tumor immune microenvironment; and reversal of therapeutic resistance. The subsections below summarize representative sources (Table 1).

**TABLE 1:** Representative flavonoids and their mechanisms against oral cancer.

TCM active metabolites	Medicinal sources	Cell lines	*In vivo* models	Targets	Effects	References
Quercetin 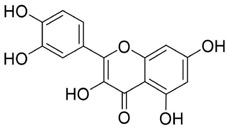	*Platycladus orientalis* (L.) Franco (Cupressaceae), *Alpinia officinarum* Hance (Zingiberaceae), *Tussilago farfara* L. (Asteraceae) et al.	YD10B, YD38	NA	↓Cyclin D1, CDK4, Bcl-2; ↑P21, Cleaved-PARP, p38 MAPK	Blocking the cell cycle in the G1 phase ↑Cell apoptosis	Son and Kim (2023)
		OSC-20, SAS, HN22	NA	↓Fibronectin, Vimentin, α-SMA et al.; ↑E-cadherin, Claudin-1	Blocking the cell cycle in the G2/M phase ↓Cell proliferation, migration, invasion capacity, EMT; ↑Cell adhesion	Kim et al. (2020)
		HSC-6, SCC-9	NA	↓HOXA10, MMP-2/-9; ↑miR-16	↓Cell viability, migratory, invasion capacity	Zhao et al. (2019)
		Tca8113, SAS	Subcutaneous injection of Tca8113 cells into nude mice	↓WNT1/β-catenin; ↑miR-22	↓Cell proliferation; ↑Cell apoptosis	Zhang et al. (2019)
		CAL-27	NA	↓CD36; ↑miR-1254	↓Cell proliferation, invasion capacity	Chen et al. (2021)
		TSCCA, SCC-15, CAL-27	NA	↓G3BP1/YWHAZ, HK2, PKM2, LDHA	↓Glucose uptake, glycolysis, cell proliferation	Hu et al. (2023b)
EGCG 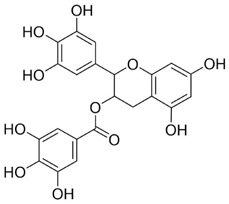	*Senegalia catechu* (L.f.) P.J.H.Hurter and Mabb. (Fabaceae)	HSC-3	Subcutaneous injection of HSC-3 cells into nude mice	↑Caspase-3/-7, Cleaved-PARP	Blocking the cell cycle in the G1 phase ↓Cell viability, proliferation; ↑Cell apoptosis	Yoshimura et al. (2019)
		Tu212, MDA686TU, SqCC/Y1 (Y1), MSK-Leuk1	Subcutaneous injection of Tu212 cells into nude mice	↓AKT/mTOR; ↑Caspase-3, Cleaved-PARP	↓Cell proliferation; ↑Cell apoptosis	Amin et al. (2021)
		CAL-27, SCC-15	NA	↓p-Akt, p-Erk, Bcl-2, Vimentin et al.; ↑Bax, Cleaved-PARP, E-cadherin	↓Cell proliferation, migratory, invasion capacity; ↑Cell apoptosis	Li et al. (2018)
		SSC4	NA	↓CASP8, MYC, TP53; ↑BAD, BAK, FAS et al.	↓Cell proliferation; ↑Cell apoptosis, autophagy	Irimie et al. (2015)
		KBV200	Subcutaneous injection of KBV200 cells into nude mice	↓VEGF	↓Angiogenesis, Cellular growth	Chen et al. (2020a)
Scutellarin 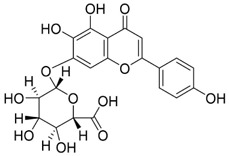	*Erigeron breviscapus* (Vaniot) Hand.-Mazz. (Asteraceae) *Scutellaria baicalensis* Georgi (Lamiaceae), *Scutellaria barbata* D. Don (Lamiaceae)	SAS	Subcutaneous injection of SAS cells into nude mice	↓MMP-2/-9, Integrin αvβ6, c-JUN	↓Cell proliferation, migratory, invasion capacity; ↑Cell apoptosis	Li et al. (2013)
		HSC-4, SAS	NA	↓Integrin αvβ6; ↑E-cadherin, Desmoplakin	↓Cell viability, proliferation, invasion capacity; ↑Cell apoptosis, cell adhesion	Li et al. (2010)
Licochalcone A 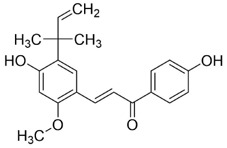	*Glycyrrhiza uralensis* Fisch. ex DC. (Fabaceae)	HN22, HSC-4	NA	↓Sp1, Cyclin D1et al.; ↑Bax, Cleaved-PARP et al.	↓Cell proliferation; ↑Cell apoptosis	Cho et al. (2014)
		FaDu	Subcutaneous injection of FaDu cells into nude mice	↑p-ERK1/2, p-p38 MAPK, Caspase-3 et al.	↑Cell apoptosis	Park et al. (2015)
		KB	NA	↑ERK and p38 MAPK, Caspase-3/-8 et al.	↑Cell apoptosis	Kim et al. (2014)
Genistein 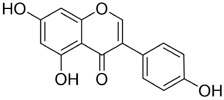	*Sophora tonkinensis* Gagnep. (Fabaceae)	NA	DMBA-induced hamster cheek pouch carcinoma model	NA	Blocking the cell cycle in the G1 phase ↓DNA synthesis/proliferation/frequency of aneuploidy; ↑Cell apoptosis	Hussein et al. (2022)
		HSC-3	Subcutaneous injection of HSC-3 cells into nude mice	↓MMP-2, VEGF mRNA	↓Cell migratory, invasion capacity, angiogenesis	Myoung et al. (2003)
Naringenin 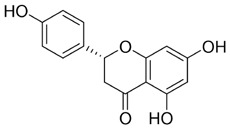	*Typha angustifolia* L. (Typhaceae), *Citrus* × *aurantium* L. (Rutaceae)	SCC-25	NA	↓Cyclin D1, AKT/ERK	Blocking the cell cycle in the G0/G1 or G2/M phase ↓Cell proliferation, migratory, invasion capacity	Maggioni et al. (2014)
		SCC-25, SCC-4	NA	↑GRP78, GRP94, Calpain I, Calpain II, Beclin 1, ATG5, LC3-II	↓Cell proliferation, mitochondrial membrane potential; ↑ROS, intracellular calcium concentration, cell apoptosis and autophagy	Liu et al. (2022a)
		NA	Subcutaneous injection of NR-S1/SCC VII (SCC7) cells into C3H/HeNCrl mice	↑CD169 + macrophages, IL-12, CXCL10	↑the infiltration and activation of CTL	Kawaguchi et al. (2022)
Nobiletin 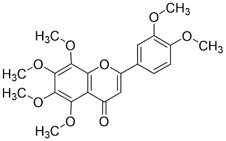	*Curcuma longa L.* (Rutaceae)	Ca9-22, HSC-3, TSC-15	NA	↑PARP, Caspase-3, γH2AX et al.	↑ROS, DNA damage, cell apoptosis	Yang et al. (2020)
		Tca8113, CAL-27	Subcutaneous injection of Tca8113/CAL-27 cells into nude mice	↓PKA/CREB	Blocking the cell cycle in the G1 phase ↓Cell proliferation, mitochondrial function, glucose consumption, pyruvate dehydrogenase activity; ↑Lactic acid and pyruvic acid	Lin et al. (2020a)
Morin 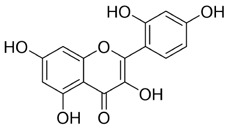	*Morus alba* L. (Moraceae)	CAL-27	NA	↓CTGF, CYR61, ANKRD; ↑p-MST1, p-MOB1, p-YAP et al.	Blocking the cell cycle in the G1 phase ↓Cell proliferation, invasion capacity	Ji et al. (2018)

#### 3.1.1 Quercetin

**FIGURE 4:**
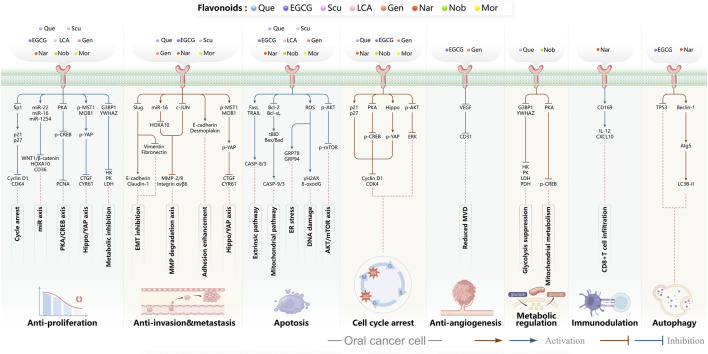
Mechanism of Flavonoid Metabolites from Traditional Chinese Medicine in Combating Oral Cancer. Flavonoids—quercetin (Que), Epigallocatechin-3-gallate (EGCG), Scutellarin (Scu), Licochalcone A (LCA), Genistein (Gen), Naringenin (Nar), Nobiletin (Nob), Morin (Mor)—primarily exert anti-oral cancer effects through mechanisms including antiproliferation, anti-migration and anti-invasion, apoptosis induction, cell cycle arrest, anti-angiogenesis, metabolic and immune regulation, and autophagy induction.

Quercetin (Que) has been demonstrated to exhibit a multifaceted array of biological activities, including antioxidant, immunoregulatory, and cardioprotective properties (Di Petrillo et al., 2022; Zhou et al., 2022; Zhang et al., 2023). In recent years, a multitude of studies have demonstrated that Que exerts pronounced inhibitory effects against various malignancies, including breast, lung, and colon cancers (Qiu et al., 2019; Lin R. et al., 2020; Guo et al., 2021). Its antitumor mechanisms involve multiple signaling pathways, encompassing the restriction of cell proliferation, suppression of invasion and metastasis, regulation of the cell cycle, induction of apoptosis, inhibition of tumor neovascularization, and modulation of autophagy (Tang et al., 2020). It is noteworthy that Que also exhibits significant anticancer potential in the context of oral cancer (Figure 4).

Regarding cell-cycle regulation, Que has been shown to impede the proliferative progression of oral cancer cells through multiple molecular pathways. Preliminary studies have demonstrated that Que triggers apoptosis in YD10B tongue squamous cell carcinoma (TSCC) cells and YD38 mandibular gingival squamous cell carcinoma cells. This process is associated with thevia activation of the p38 MAPK signaling pathway. This process is marked by the downregulation of Cyclin D1 and CDK4, and the upregulation of the CDK inhibitor p21, leading to G1-phase arrest (Son and Kim, 2023). Studies also show that Que can trigger G2/M-phase arrest, suppressing the growth of human tongue squamous cell carcinoma cell lines (OSC-20 and SAS) and human laryngeal squamous cell carcinoma cells (HN22) (Kim et al., 2020). The findings collectively suggest that quercetin can impede tumor progression in oral cancer by modulating cell-cycle progression.

Simultaneously, Que suppresses the migratory and invasive behavior of oral cancer cells through multiple molecular mechanisms. Firstly, Que suppresses the migratory and invasive abilities of HN22 cells and human TSCC lines (OSC-20, SAS, HSC-6, and SCC-9) through suppression of MMP-2 and MMP-9 expression (Zhao et al., 2019; Kim et al., 2020). Secondly, microRNAs (miRNAs) have been identified as key molecular targets of Que (Kim et al., 2019). By modulating miRNA expression, Que orchestrates multiple cellular functions, including proliferation, migration, and apoptosis (Mohr and Mott, 2015). Studies have shown that Que upregulates miR-16, which suppresses HOXA10, thereby decreasing cell viability and migration/invasion in HSC-6 and SCC-9 cells (Zhao et al., 2019). Thirdly, Que upregulates miR-22, downregulate the Wnt/β-catenin molecular pathway, and induce apoptosis in human TSCC cell lines Tca8113 and SAS (Zhang et al., 2019). It also elevates miR-1254 while downregulating CD36, dose-dependently inhibiting proliferation and invasion in CAL-27 cells (Chen et al., 2021). Furthermore, Que blocks the EMT process via regulation of key molecular targets such as E-cadherin, claudin-1, vimentin, fibronectin, α-SMA, and Slug, thereby reducing the migratory and invasive potential of OSC-20, SAS, and HN22 cells (Kim et al., 2020).

Aberrant energy metabolism is a hallmark of tumor progression. Ras GTPase-activating protein-binding protein 1 (G3BP1) is overexpressed in several malignancies, including OSCC, whereas tyrosine 3-monooxygenase/tryptophan 5-monooxygenase activation protein zeta (YWHAZ) functions as a pivotal signaling hub in cancer (Cao et al., 2023). Que reduces glucose uptake and glycolytic activity in human TSCC cell lines (TSCCA, SCC-15, and CAL-27) by inhibiting the G3BP1/YWHAZ signaling axis. This inhibition diminishes the metabolic energy supply of tumor cells, thereby suppressing their proliferation (Hu M. et al., 2023).

Network pharmacology analyses suggest that Que may target multiple key proteins (e.g., AKT1 and PIK3R1) via the PI3K/AKT signaling pathway (Dong et al., 2023). These findings offer new insights into using Que as a treatment for oral cancer. The above studies indicate that Que, a purified, plant-derived monomer, can inhibit the proliferation, migration, and invasion of OSCC cells *in vitro*. This process involves glycolytic reprogramming, EMT and miRNA regulation. However, it should be noted that the current evidence is largely based on *in vitro* experiments, with only a few studies incorporating xenograft validation. Common treatment concentrations are primarily within the 10–160 μM range, and sometimes higher. While some studies have observed dose-dependent effects, standardized IC_50_ parameters, definitions of minimum effective concentrations, and positive drug controls are generally lacking. *In vivo* validation, pharmacokinetic studies, and toxicity assessments are also insufficient. Therefore, clinical translation requires further confirmation through optimization of safety and delivery in more relevant model systems. Despite promise, clinical translation is hampered by low solubility, poor bioavailability, and suboptimal pharmacokinetics. In order to address these limitations, the use of nanoformulations, such as nanostructured lipid carriers (NLCs), has been proposed as a strategy to enhance stability, targeting efficiency, and cellular uptake (Chaudhari et al., 2021). Concurrently, rational structural modification has the potential to yield derivatives with enhanced pharmacokinetic profiles and augmented anticancer efficacy.

#### 3.1.2 Epigallocatechin-3-gallate

Epigallocatechin-3-gallate (EGCG) is a natural polyphenolic metabolite found in tea leaves and other botanical sources. Its antioxidant and cancer-suppressive activities have been extensively documented (Peng et al., 2025). Recent basic and preclinical studies have demonstrated significant activity of EGCG against oral cancer (Figure 4). Reported mechanisms include cell-cycle regulation, apoptosis and autophagy activation, and suppression of migration, invasion, and tumor angiogenesis. (Chen L. et al., 2020; Marín et al., 2023).

EGCG regulates cell-cycle progression, reducing the viability of oral cancer cells and inducing G1-phase arrest (Yoshimura et al., 2019; Amin et al., 2021). Mechanistically, the drug upregulates B-cell translocation gene 2 (BTG2) and modulates MAPK signaling, thereby suppressing *in vitro* proliferation and *in vivo* tumor growth (Lee et al., 2015). With respect to apoptosis regulation, EGCG activates caspase-3/7 and promotes cleavage of poly (ADP-ribose) polymerase (PARP), thereby impairing DNA repair and triggering DNA fragmentation. The cascade induces apoptosis in a series of cell lines, including those from invasive and metastatic tongue squamous cell carcinoma (HSC-3, MDA686TU), hypopharyngeal carcinoma (Tu212), laryngeal carcinoma (SqCC/Y1), and oral leukoplakia (MSK-Leuk1). This process is accompanied by a significant decrease in Ki-67 expression (Yoshimura et al., 2019; Amin et al., 2021). Furthermore, EGCG modulates JNK phosphorylation and interacts with Hippo-TAZ signaling, reducing LATS1 and TAZ protein levels and thereby regulating proliferation and apoptosis in CAL-27 and SCC-15 cells (Li et al., 2018). In addition, EGCG promotes autophagy by regulating autophagy-related pathways, leading to cell death in OSCC cells like SSC4, suggesting its role in determining cell fate (Irimie et al., 2015). EGCG attenuates the metastatic potential of CAL-27 and SCC-15 cells by engaging MAPK and Hippo-TAZ pathways (Li et al., 2018). It also suppresses neovascularization by downregulating VEGF, in combination with vincristine sulfate (VCR), EGCG can also enhance inhibition of tumor growth and angiogenesis in multidrug-resistant KBV200 OSCC cells (Chen L. et al., 2020). In summary, EGCG exerts its effects through the coordinated regulation of multiple targets and signaling networks in oral cancer.

EGCG typically inhibits the proliferation of and induces apoptosis in oral cancer cells *in vitro* at concentrations ranging from 25 to 200 μM. However, significant effects often require concentrations of ≥80–100 μM, and there is no consistent IC_50_ value. Nude mouse xenograft studies have suggested that oral administration of 125 mg/kg or intraperitoneal injection of 75 mg/kg of EGCG reduces tumor volume by approximately 45% (Yoshimura et al., 2019). In multidrug-resistant models, combining EGCG with vincristine at 10–40 mg/kg enhances chemotherapy sensitivity (Chen L. et al., 2020). Overall, however, existing *in vivo* doses are generally high, and systematic pharmacokinetic, tissue exposure, and long-term safety data supporting clinical feasibility are lacking. Further validation is required for clinical translation.

#### 3.1.3 Scutellarin

Scutellarin (Scu) is a flavonoid metabolite predominantly found in *Erigeron* and *Scutellaria* species. The substance exhibits a wide range of pharmacological activities, including antibacterial, antiviral, anticancer, and neuroprotective effects (Xie et al., 2024). Scu treats oral cancer in several ways, including stopping the growth of tumor cells, regulating the cell cycle, causing tumor cells to die, and preventing new blood vessels from forming (Bayat et al., 2024) (Figure 4).

Overexpression of integrin αvβ6 predicts poor prognosis and high metastatic potential in several malignancies (Bandyopadhyay and Raghavan, 2009). Both *in vivo* and *in vitro* studies have demonstrated that Scu effectively inhibits the invasion and proliferation of human squamous cell carcinoma (SAS cells) by downregulating the expression of MMP-2, MMP-9, and integrin αvβ6, while simultaneously inducing apoptosis and exerting potent antitumor effects (Li et al., 2013). The epithelial adhesion molecule E-cadherin is critical for maintaining epithelial integrity and constraining metastasis. Treatment of TSCC cells (HSC-4, SAS) with graded concentrations of Scu (3–15 nM) increases E-cadherin expression and decreases integrin αvβ6 levels, enhancing cell adhesion and suppressing proliferation and migration in OSCC cells (Li et al., 2010).

In summary, Scu exhibits potent anti-invasive properties in the OSCC model. *In vitro* experiments revealed that, at a concentration of 15 nM, scutellarin reduced the migration of HSC-4 and SAS cells by approximately 44%–46% (Li et al., 2010). This effect was mediated, at least in part, by downregulating integrin αvβ6 and MMP-2/9 expression and upregulating E-cadherin. This process inhibits OSCC invasion, migration, and proliferation while promoting apoptosis. *In vivo*, oral administration of 10–20 mg/kg in SAS xenograft models achieved tumor inhibition rates of 55%–68.5% at lower doses than most natural metabolites (Li et al., 2013). However, the mechanism primarily resides at the integrin/MMP expression level, necessitating more rigorous causal validation and standardized control studies, as exposure bridging between nanomolar effects *in vitro* and mg/kg dosing *in vivo* remains absent.

#### 3.1.4 Licochalcone A

Licochalcone A (LCA) belongs to the flavonoid class isolated from the root of *Glycyrrhiza uralensis* Fisch. ex DC. (Fabaceae) commonly used in TCM. Extensive studies have shown that LCA exhibits broad pharmacological activities, including anticancer, anti-inflammatory, antibacterial, antioxidant, neuroprotective, and antiparasitic effects. Anticancer actions encompass the suppression of tumor growth, migration, and invasion, induction of apoptosis, modulation of the tumor immune microenvironment, and reversal of drug resistance (Li M. T. et al., 2022) (Figure 4).

The Specificity Protein (Sp) transcription factor family is correlated with unfavorable prognosis and modulates transformation, proliferation, migration, and invasion by controlling protein-coding genes and interacting with miRNAs and long non-coding RNAs (lncRNAs) (Safe, 2023). Among these, Sp1 is a critical oncogenic factor in multiple tumor types. LCA downregulates Sp1 expression in a dose- and time-dependent manner and modulates Sp1-linked signaling. In oral cancer models, LCA (10–40 μM) treatment of HN22 and HSC-4 cells reduces Sp1, induces apoptosis, and inhibits proliferation (Cho et al., 2014). Consistently, similar Sp1-dependent anticancer effects have been observed in renal cell carcinoma models (Tseng et al., 2023). Conversely, apoptosis-related ligands, including tumor necrosis factor–related apoptosis-inducing ligand (TRAIL) and Fas ligand (FasL), are key mediators of caspase-dependent death signaling. The IC_50_ in FaDu cells is approximately 100 μM, LCA promotes ERK1/2 and p38 MAPK phosphorylation, induces TRAIL expression, and markedly enhances pro-apoptotic activity. LCA (10 mg/kg) also suppresses xenograft growth by activating caspase-3 (Park et al., 2015). Moreover, the IC_50_ in KB cells is approximately 50 μM, LCA upregulates FasL and activates caspase-3 and caspase-8, triggering a caspase cascade that culminates in apoptosis (Kim et al., 2014).

Overall, LCA inhibits proliferation and survival through Sp1 downregulation and associated pathway modulation, while concomitantly upregulating TRAIL and FasL to activate caspase-dependent apoptosis. However, effective concentrations are generally still elevated, and there is insufficient pharmacokinetic bridging between *in vitro* and *in vivo* exposure data. Additionally, oral cancer research has largely focused on subcutaneous xenografts and a limited number of cell lines. These findings suggest that clinical translation requires further validation using *in situ* models, an assessment of dose feasibility, and support for standardized formulation development.

#### 3.1.5 Genistein

Genistein (Gen) is an isoflavone predominantly found in *Sophora tonkinensis* Gagnep. (Fabaceae) It exhibits broad pharmacological properties, involving antioxidant, anticancer, antibacterial, antiviral, and anti-diabetic effects (Sharifi-Rad et al., 2021). Recent studies have demonstrated significant antitumor potential across multiple cancers: Gen suppresses breast-cancer growth and metastasis (Mukund, 2020), induces mitochondrial-mediated apoptosis in non–small-cell lung cancer (Chan et al., 2022), and demonstrates activity in colorectal, prostate, and ovarian cancers (Sahin et al., 2019; Chen X. et al., 2020; Shafiee et al., 2022), highlighting its promise for the prevention and treatment of cancer.

In oral cancer (Figure 4), topical application of Gen to the oral mucosa reduces abnormal proliferation and DNA aneuploidy, thereby limiting invasion and metastasis in a hamster buccal-pouch carcinoma model (Hussein et al., 2022). Subsequent *in vitro* and *in vivo* studies further reveal that Gen inhibits angiogenesis and tumor-cell invasion, exerting anticancer effects against HSC-3 cells (Myoung et al., 2003). However, the clinical utility of Gen is constrained by low bioavailability. To address this, nanoformulated Gen has been shown to alleviate epigenetic transcriptional repression and selectively induces apoptosis in the human HPV-positive oropharyngeal carcinoma line JHU-011, achieving potent activity under low-toxicity conditions (Dev et al., 2021).

In summary, Gen primarily exerts its effects in OSCC by inhibiting processes related to invasion and angiogenesis. At an *in vitro* concentration of approximately 27.3 μg/mL, Gen reduces MMP-2 activation and VEGF expression (Myoung et al., 2003). However, its limited improvement in tumor burden in animal xenograft models suggests that its antitumor effects may be insufficient for independent therapeutic application. In chemoprevention models, oral administration of 20 mg/kg/day delays lesion progression (Hussein et al., 2022). Meanwhile, nanoparticle delivery systems (e.g., lactalbumin-GLNPs) enhance the cytotoxic effects at around 40 μM by improving solubility and cellular uptake (Dev et al., 2021). These findings suggest that Gen’s future value may lie in optimizing bioavailability and developing combination therapy strategies.

#### 3.1.6 Naringenin

Naringenin (Nar) is a naturally occurring bioactive flavonoid predominantly found in citrus fruits and in TCM such as *Typha angustifolia* L. (Typhaceae) and *Citrus* × *aurantium* L. (Rutaceae). It exhibits a broad spectrum of pharmacological activities, including anti-inflammatory, antiviral, antidiabetic, neuroprotective, and immunomodulatory activities (Den Hartogh and Tsiani, 2019; Nouri et al., 2019; Tutunchi et al., 2020). In cancer therapy, Nar influences tumor initiation and progression via multiple mechanisms, including inhibition of tumor proliferation, migration, and invasion; induction of apoptosis; attenuation of inflammation; regulation of autophagy; and suppression of angiogenesis (Stabrauskiene et al., 2022).

In OSCC (Figure 4), Nar inhibits migration and invasion of TSCC (SCC-25) cells while inducing apoptosis and causing cell-cycle arrest at G0/G1 and G2/M phases (Maggioni et al., 2014). Mechanistically, The IC_50_ values of naringenin in SCC-4 and SCC-25 cells were 232 μM and 132 μM, respectively, with a trend toward clonogenic inhibition observed at concentrations between 25–50 μM.Nar modulates endoplasmic reticulum (ER) stress-related family proteins in both cell types, elevates intracellular Ca^2+^ levels, leads to mitochondrial depolarization and excessive accumulation of reactive oxygen species (ROS), thereby promoting apoptosis and autophagy while suppressing proliferation and metastatic dissemination (Liu J. F. et al., 2022). With respect to tumor immunity, intraperitoneal injection of 40 mg/kg naringin can enhance T cell-mediated antitumor immune responses by activating CD169^+^ macrophages in subcutaneous tumor models established with the rat OSCC cell line NR-S1 and the mouse squamous cell carcinoma line SCC7 (Kawaguchi et al., 2022). This activation correlates with favorable tumor outcomes, suggesting that Nar may represent a promising adjuvant candidate for cancer immunotherapy.

Overall, Nar has demonstrated potential in oral cancer research, but there is significant uncertainty regarding translational evidence. *In vitro* experiments typically require concentrations of 10–250 μM to induce ROS/ER stress-related apoptosis (Liu J. F. et al., 2022). Its IC_50_ remains in the micromolar range of 132–232 μM, which suggests relatively limited direct cytotoxic efficacy and questionable clinical tissue exposure feasibility. In contrast, scutellarin inhibits migration at nanomolar concentrations, while EGCG and genistein typically exert effects within the tens of micromolar range. These results demonstrate significant variations in the potency of different natural products. *In vivo*, 40 mg/kg administration suppresses tumor growth and enhances CD8^+^ T cell infiltration (Kawaguchi et al., 2022). However, the absence of systematic pharmacokinetics (PK) and long-term safety evaluations leaves unclear whether its immune effects are accompanied by non-specific inflammatory activation. Furthermore, this mechanism relies on the activation of CD169^+^ macrophages in lymph nodes; however, the causal chain linking tumor antigen presentation to CTL activation is incomplete. This suggests that the universality of this immune regulation requires further validation. Consequently, naringenin is more likely to serve as an immunomodulatory adjuvant candidate. Future clinical translation should be based on establishing an exposure window, confirming safety, and evaluating its value in combination therapy for sensitization.

#### 3.1.7 Nobiletin

Nobiletin (Nob) is a polymethoxylated flavonoid abundant in citrus fruits. Nob exhibits diverse biological activities, including hepatoprotective, anti-inflammatory, anticancer, neuroprotective, and antidiabetic effects. Reported anticancer mechanisms include induction of apoptosis and cell-cycle arrest, suppression of migratory and invasive capacities, attenuation of multidrug resistance, and modulation of immune responses (Zhang et al., 2021). Growing evidence highlights its antitumor potential in oral cancer (Figure 4).

In terms of inhibiting cell proliferation and inducing apoptosis, Nob (25–100 μM) suppresses viability of human gingival squamous cell carcinoma (Ca9-22) and tongue squamous cell carcinoma (HSC-3, SCC-15), increases ROS and DNA damage, and thereby promotes apoptosis. The effects on normal human oral epithelial cells (HOECs) are comparatively minor, indicating selective cytotoxicity toward tumor cells (Yang et al., 2020). With respect to tumor cell metabolism and mitochondrial regulation, Nob disrupts mitochondrial function via protein kinase A–cAMP response element–binding protein (PKA–CREB) signaling, reducing glucose consumption and pyruvate dehydrogenase activity. The resultant accumulation of lactate and pyruvate induces G1-phase arrest and inhibits proliferation in Tca8113 and CAL-27 cells. In addition, Nob is essentially ineffective below 50 μM. At concentrations of 50–150 μM *in vitro* or a dose of 40 mg/kg *in vivo*, it significantly suppresses OSCC tumor growth, supporting potent anticancer activity (Lin C. X. et al., 2020).

Collectively, current evidence indicates that In oral cancer *in vitro* models, Nob requires concentrations of ≥50–100 μM to further inhibit proliferation and induce ROS/DNA damage and G1 arrest (Lin C. X. et al., 2020; Yang et al., 2020). *In vivo*, an effective concentration of 40 mg/kg inhibits xenograft tumour growth (Lin C. X. et al., 2020); however, the mitochondrial toxicity window in normal cells remains unclear and efficacy may be influenced by the cAMP metabolic background. Therefore, it is more suitable as a candidate for adjuvant therapy targeting metabolic regulation than as a direct, highly effective monotherapy.

#### 3.1.8 Morin

Morin (Mor) is a dietary flavonoid present in traditional Chinese medicinal botanical drugs, including *Morus alba* L. (Moraceae). It exhibits diverse pharmacological properties, such as antioxidant, anti-inflammatory, anticancer, neuroprotective, and cardioprotective effects (Thakur et al., 2020; Shyma and Mini, 2022). Moreover, Morin mitigates drug-induced toxicity, highlighting its potential utility in combination chemotherapy (Kucukler et al., 2020; Çomaklı et al., 2022). Anticancer activities involve multiple mechanisms, including inhibition of proliferation, metastasis, and invasion; regulation of the cell cycle; induction of apoptosis; suppression of angiogenesis; and modulation of miRNA expression (Thakur et al., 2020).

The Hippo pathway regulates tissue homeostasis by controlling cell proliferation and apoptosis. Its core effector, Yes-associated protein (YAP), acts as a transcriptional coactivator whose activity is restricted by Hippo-mediated phosphorylation (Harvey et al., 2013). Studies have shown that Mor suppresses proliferation and migration of CAL-27 cells (Figure 4), primarily by activating Hippo signaling and inhibiting YAP nuclear translocation and YAP-driven transcription, thereby exerting antitumor effects against TSCC *in vitro* (Ji et al., 2018). Although morin, a purified flavonoid monomer, inhibits proliferation and activates the Hippo/YAP pathway in CAL-27 cells, the existing evidence is lacking in several respects. Its primary effective concentration in oral cancer cells is in the range of 50–150 μM, which suggests that its direct antitumour potency is limited. Furthermore, there is a lack of *in vivo* evidence, and its clinical application may require delivery optimisation due to its solubility and bioavailability.

### 3.2 Terpenoids

Terpenoids constitute a large and structurally diverse class of bioactive metabolites in TCMs. They display diverse pharmacological properties, encompassing anti-inflammatory, antimicrobial, and anticancer effects. In oral cancer, terpenoids act through multifaceted mechanisms, such as inducing cell-cycle arrest, triggering mitochondrial-dependent apoptosis, regulating autophagy, inhibiting angiogenesis, and remodeling the tumor immune microenvironment. Notable representatives that have been extensively studied include triptolide, ursolic acid, and oridonin. The subsections below summarize their sources, principal molecular targets and pathways, and key preclinical evidence (Table 2).

**TABLE 2:** Representative terpenoids and their mechanisms against oral cancer.

TCM active metabolites	Medicinal sources	Cell lines	*In vivo* models	Targets	Effects	References
Triptolide 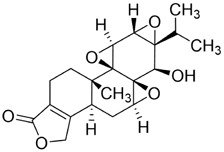	*Tripterygium wilfordii* Hook.f. (Celastraceae)	SCC-25, CAL-27	Subcutaneous injection of SCC-25 cells into nude mice	↓JUN, MAPK8, STAT3, VEGFA, IL2, CXCR4, PTGS2, IL4	↓Cell proliferation, invasion capacity	Hao et al. (2023b)
		SAS	PDTX model Subcutaneous injection of SAS cells into NOD/SCID mice	↓IFN-γ, JAK2/STAT1, PD-L1	↓Cell proliferation; ↑Immune cell infiltration	Kuo et al. (2021)
		SAS, SCC-25, HSC-3	PDTX model	↓DcR3, MTA1	↓Cell proliferation	Yang et al. (2019a)
		CAL-27, WSU-HN6	Subcutaneous injection of WSU-HN6 cells into nude mice Orthotopic xenograft model with WSU-HN6 cells	↓METTL3, SLC7A11	↓Cell proliferation, migratory, invasion capacity, the rate of lymphatic metastasis	Xu et al. (2021)
Ursolic acid 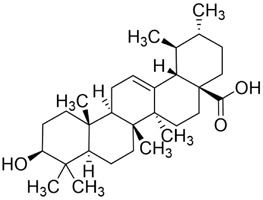	*Scleromitrion diffusum* (Willd.) R.J.Wang (Rubiaceae), *Prunella vulgaris* L. (Lamiaceae), *Ligustrum lucidum* W.T.Aiton (Oleaceae),et	Ca9-22 SCC-2095	NA	↓Akt/mTOR/NF-κB, ERK, p38 MAPK, MMP-2; ↑Cleaved-PARP, Caspase-7/9, p62, the transformation of LC3B-II	↓Cell proliferation, migratory, invasion capacity; ↑DNA damage, Cell apoptosis and autophagy	Lin et al. (2019)
		CAL-27	NA	↓Bcl-2, Bcl-xL, Bax, p-AKT (Ser473), p-BAD (Ser136); ↑Caspase-3/-9, BAD	↓Mitochondrial membrane potential; ↑ROS, Cell apoptosis	Chen et al. (2018)
Oridonin 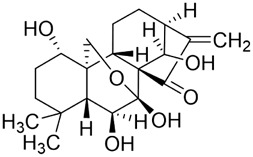	*Gongronemopsis tenacissima* (Roxb.) S.Reuss, Liede and Meve (Apocynaceae)	CAL-27	NA	↓BCL2L2, BIRC3, BCL6, TNFSF10, Bcl-w, cIAP2, TRAIL; ↑Bax, AIFM2, Cleaved caspase-3	↓Cell proliferation; ↑Cell apoptosis	Wu et al. (2023a)
		WSU-HN4, WSU-HN6, CAL-27	Subcutaneous injection of WSU-HN6 cells into nude mice	↓Cdc25C, Cdc2, Cyclin B1; ↑P53, Caspase-3/-9 et al.	Blocking the cell cycle in the G2/M phase ↑Cell apoptosis	Wang et al. (2017)
		SCC-25, UM1, UM2, HSC-3, CAL-27	Subcutaneous injection of SCC-25 cells into nude mice	↓Cyclin B1, Bcl-2, PI3K/Akt; ↑p-CDK1, Bax, Bax/Bcl-xl, Caspase-3/-9, Cleaved-PARP et al.	Blocking the cell cycle in the G2/M phase ↓Cell proliferation; ↑Cell apoptosis	Yang et al. (2018)
Artemisinin 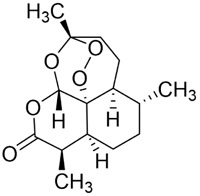	*Artemisia annua* L. (Asteraceae)	CAL-27, HN31	Subcutaneous injection of CAL-27 cells into nude mice	↓VEGF, IL-8, MIF	↓Cell proliferation, Angiogenesis; ↑Vascular normalization	Yu et al. (2024)
		A-253, SCC-25	NA	↓Bcl-2, Bcl-xL; ↑Caspase-3, PARP1, Bax, Bim, Bad, Bak	Blocking the cell cycle in the G0/G1 phase ↓Cell proliferation; ↑ROS, Cell apoptosis	Xiao et al. (2022)
Cantharidin 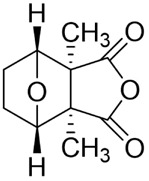	*Mylabris phalerata* Pallas (Coleoptera: Meloidae)	Tca8113	NA	↓miR-214, Bcl-2; ↑P53, Bax	↓Cell proliferation; ↑Cell apoptosis	Tian et al. (2015)
		SAS, CAL-27, SCC-4	NA	↓Bcl-2, JNK-regulated mitochondrial transmembrane potential; ↑p-eIF-2α, CHOP, Cleaved-caspases-9/-7/-3 et al.	↑Cytotoxic effects, ER stress, Cell apoptosis	Su et al. (2016)

#### 3.2.1 Triptolide

Triptolide (TPL) is an epoxy diterpenoid metabolite isolated from the TCMs *Tripterygium wilfordii* Hook. f. (Celastraceae). Beyond its well-known anti-inflammatory and antirheumatic immunomodulatory activities, recent studies have identified TPL as a broad-spectrum anticancer agent with activity across multiple solid tumors. Reported mechanisms include promotion of apoptosis, induction of cellular senescence, regulation of the cell cycle, activation of autophagy, and suppression of tumor-cell proliferation, migration, and invasion. TPL has also shown therapeutic potential in neurodegenerative disease models, underscoring its diverse pharmacological profile (Gao et al., 2021).

**FIGURE 5:**
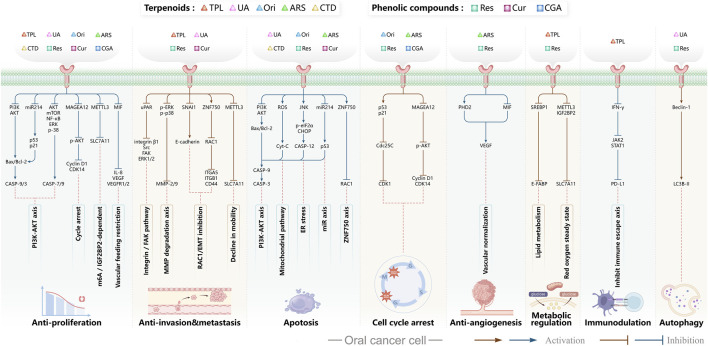
Mechanisms of Action of Terpenoids and Phenolic Metabolites from Traditional Chinese Medicine Extracts Against Oral Cancer. Terpenoids: Triptolide (TPL), Ursolic acid (UA), Oridonin (Ori), Artemisinin (ARS), Cantharidin (CTD), and phenolic metabolites: Resveratrol (Res), Curcumin (Cur), Chlorogenic acid (CGA), primarily exert their anti-oral cancer effects through mechanisms including antiproliferation, anti-migration and anti-invasion, apoptosis induction, cell cycle arrest, anti-angiogenesis, metabolic and immune regulation, and autophagy induction.

In oral cancer (Figure 5), TPL exerts multitarget and multipathway effects. Network pharmacology predicts interactions with 17 OSCC-associated targets, with predominant modulation of JAK-STAT and MAPK pathways (Hao P. et al., 2023). Consistent *in vitro* and *in vivo* evidence indicates that TPL restrains cell proliferation of SCC-25 and CAL-27 cells while downregulating key proteins including JUN, MAPK8, STAT3, TP53 and IL4 (Hao P. et al., 2023). TPL suppresses IFN-γ secretion and attenuates JAK/STAT signaling, thereby reducing PD-L1 expression in SAS tongue squamous cell carcinoma (TSCC) cells; these changes enhance immune-cell infiltration and inhibit tumor growth (Kuo et al., 2021). Regarding metastasis-associated molecules, decoy receptor 3 (DcR3)—a biomarker linked to metastasis and poor prognosis in OSCC—is suppressed by TPL in SAS, SCC-25, and HSC-3 cells and in patient-derived tumor xenografts (PDTX), leading to reduced tumor proliferation (Hsieh and Lin, 2017; Yang C. Y. et al., 2019). Moreover, TPL downregulates methyltransferase-like 3 (METTL3), which consequently decreases SLC7A11 mRNA expression and stability, curbing proliferation, migration, and invasion in CAL-27 and WSU-HN6 cells (Xu et al., 2021). Collectively, these findings indicate that TPL exerts comprehensive antitumor activity against OSCC by simultaneously regulating apoptosis, immune modulation, oncogenic signaling, and metastasis-related molecules.

Although TPL exhibits nanomolar inhibitory activity (30–100 nM) in oral cancer and significantly suppresses tumours at 0.15 mg/kg/day in PDTX models, its translation into clinical practice faces critical obstacles. Firstly, TPL is a potent cytotoxic metabolite. Despite its outstanding *in vitro* efficacy, its narrow therapeutic window and the risk of hepatotoxicity limit the feasibility of long-term administration (Gao et al., 2021). Secondly, the current *in vivo* evidence base is largely derived from subcutaneous xenografts and PDTX models. The lack of oral *in situ* models and systematic PK-exposure bridging makes it difficult to assess clinically achievable concentrations. Furthermore, studies investigating mechanisms predominantly focus on pathway nodes such as DcR3 or PD-L1 downregulation. This necessitates comparison with standardised control drugs to clarify their relative advantages. Consequently, TPL is more likely to serve as a “highly potent but highly toxic” lead scaffold. Its clinical potential will depend on future efforts to reduce toxicity and enhance efficacy through derivative design or optimisation of the delivery system.

#### 3.2.2 Ursolic acid

Ursolic acid (UA) is a pentacyclic triterpenoid metabolite abundantly present across numerous TCMs, including *Scleromitrion diffusum* (Willd.) R.J.Wang (Rubiaceae), *Prunella vulgaris* L. (Lamiaceae), and *Ligustrum lucidum* W.T.Aiton (Oleaceae). It exhibits a wide spectrum of pharmacological activities, such as anticancer, antioxidant, antibacterial, antiviral, and organ-protective effects (Tohmé et al., 2019; Chen Z. et al., 2020; Lyu et al., 2021; Ma et al., 2021; Liu K. M. et al., 2022). Growing evidence highlights UA’s anticancer potential, involving suppression of proliferation and metastasis, induction of cell-cycle arrest, activation of apoptosis and autophagy, and synergy with conventional agents (Lin et al., 2019; Wang S. et al., 2021; Li H. et al., 2022; Zhao et al., 2023).

Autophagy is a process by which macromolecules and organelles are delivered to lysosomes for degradation. In OSCC (Figure 5), autophagy has emerged as a key mechanism of UA action. UA promotes conversion of LC3B-I to LC3B-II, thereby triggering autophagy in Ca9-22 oral squamous carcinoma and SCC-2095 human laryngeal squamous carcinoma cells. These changes inhibit proliferation, induce apoptosis, and suppress migration and invasion in a dose- and time-dependent manner (Lin et al., 2019). Further investigations have shown that UA also activates the caspase-dependent apoptosis pathway. Specifically, UA activates caspase-dependent apoptosis, enhancing caspase-3 and caspase-9 activities, increasing ROS, and promoting dissipation of mitochondrial membrane potential. Concomitantly, it suppresses anti-apoptotic proteins Bcl-2 and Bcl-xL while enhances BAD and Bax, thereby reducing the viability of drug-resistant (Chen et al., 2018). Despite its pronounced anti-oral cancer potential, UA’s clinical application is limited by poor aqueous solubility and low bioavailability. To address this, researchers have developed chitosan-based UA nanoparticles (UACNPs) and evaluated their efficacy in a DMBA. Hamsters treated with DMBA plus UACNPs showed no tumor formation, exhibited only mild histopathological alterations, and maintained near-normal antioxidant status. These findings indicate that UACNPs potentiate UA’s antitumor effects, partly by scavenging excess ROS (Karthik et al., 2023).

In summary, although UA induces apoptosis and autophagy while inhibiting invasion in OSCC cells, its *in vitro* effects predominantly occur at concentrations of 100–200 μM or higher. This suggests a potential gap between therapeutic potency and clinically achievable exposure levels. Furthermore, existing mechanisms primarily involve pathway associations (e.g., Akt/mTOR, LC3B conversion), but there is no rigorous evidence of causal relationships. Chitosan nanodelivery (12.5 mg/kg) significantly reduced tumour incidence in the DMBA hamster oral carcinogenesis model, suggesting that optimising delivery may be key to realising its translational potential.

#### 3.2.3 Oridonin

Oridonin (Ori) is a phellandrene-type tetracyclic diterpenoid that has been widely investigated for its pharmacological activities, including anti-inflammatory, antiviral, antibacterial, anti-aging, and organ-protective effects (Xu et al., 2019; Yang H. et al., 2019; Yu et al., 2019; Yuan et al., 2019; Tan et al., 2021; An et al., 2022; Zhan et al., 2022; Zhong et al., 2022). Extensive research has demonstrated potent anticancer activity, encompassing suppression of cell proliferation, migration, and invasion (Du et al., 2019; Zhou et al., 2021); inhibition of EMT (Lou et al., 2019; Xu et al., 2020; Liu W. et al., 2022); induction of apoptosis (Luo et al., 2019); inhibition of angiogenesis (Li et al., 2019; Zhou et al., 2021); and modulation of tumor immune responses (Hwang and Chang, 2023; Liu et al., 2024). Recent investigations have begun to delineate Ori’s specific roles and molecular mechanisms in oral cancer (Figure 5).

First, Ori inhibits the growth of CAL-27 cells by modulating key genes (BIRC3, TNFSF10, and BCL6) through both mitochondrial and death-receptor pathways, thereby triggering apoptosis (Wu G. et al., 2023). Second, Ori (10–20 μM) induces G2/M-phase arrest in multiple OSCC lines (WSU-HN4, WSU-HN6, CAL-27, and SCC-25), accompanied by downregulation of G2/M transition proteins and activation of intrinsic apoptotic signaling (Wang et al., 2017). Finally, in primary and recurrent floor-of-mouth carcinoma lines (UM1, UM2) and TSCC lines (SCC-25, HSC-3, CAL-27), The IC_50_ values after 48 h of oridonin treatment were as follows: SCC25: 9.07 μM; UM1: 8.15 μM; HSC3: 15.39 μM. Ori enhances apoptosis and cell-cycle arrest by inhibiting the PI3K/AKT pathway, thereby amplifying its antitumor effects (Yang et al., 2018). Collectively, Ori acts in oral cancer by suppressing proliferation, enforcing cell-cycle arrest, and activating apoptotic pathways, with PI3K/AKT inhibition as a central axis.

Although triptolide and oridonin are both natural products of the diterpene class, they exhibit distinct differences in toxicity and mechanisms of action. Triptolide demonstrates nanomolar potency in OSCC and has the potential to modulate immune checkpoints. However, its therapeutic window is narrow, it carries significant hepatotoxicity risks and its translation is therefore more reliant on toxicity reduction strategies. In contrast, oridonin primarily exhibits micromolar activity (IC_50_ = 8–15 μM) with mechanisms centred on cell cycle arrest, mitochondrial apoptosis and PI3K/Akt inhibition. It enriches TNF/NF-κB-related apoptotic networks, but there is relatively limited evidence for immune checkpoint modulation. Consequently, Oridonin emerges as a promising candidate for oral cancer treatment, offering moderate potency and a strong focus on apoptosis.

#### 3.2.4 Artemisinin

Artemisinin (ARS) is a bioactive sesquiterpene lactone molecule isolated from the *Artemisia annua* L. (Asteraceae). It is widely recognized as a first-line antimalarial drug. Beyond its potent antimalarial efficacy, ARS and its derivatives show broad therapeutic potential across infectious and non-communicable diseases (Feng et al., 2020). Reported anticancer mechanisms encompass induction of apoptosis, regulation of autophagy, cell-cycle arrest, promotion of ROS generation and oxidative stress, inhibition of angiogenesis, induction of DNA damage, and activation of ferroptosis (Zhu S. et al., 2021; Zeng et al., 2023).

Macrophage migration inhibitory factor (MIF) is a pro-tumorigenic cytokine that signals through CD74 to activate pathways driving cell proliferation, invasion, and angiogenesis. Elevated MIF has been reported in OSCC is associated with enhanced malignancy (Kang et al., 2018; Klemke et al., 2021). ARS suppresses malignant phenotypes in oral cancer–related cells such as CAL-27 and HN31 (Figure 5). ARS also inhibits proliferation, migration, and invasion while downregulating angiogenesis-associated genes (VEGF, IL8, and MIF), thereby reducing angiogenesis, promoting vascular normalization, and restraining tumor growth. Notably, the optimal “vascular normalization window” was observed with an intraperitoneal dose of 50 mg/kg (Yu et al., 2024). Furthermore, ARS induces apoptosis in oral cancer models. It inhibits proliferation of salivary gland carcinoma (A-253) and SCC-25 cells in a concentration-dependent manner, causing G0/G1-phase arrest, elevating intracellular ROS, activating mitochondrial apoptotic signaling, and ultimately triggering apoptosis (Xiao et al., 2022). Collectively, ARS and its derivatives suppress oral-cancer proliferation and invasion via direct cytotoxic effects and microenvironmental modulation. By downregulating MIF and remodeling angiogenesis, ARS exerts multifaceted anticancer activity and shows strong promise for clinical translation in oral cancer therapy.

Compared to other terpenoids such as triptolide and oridonin, ARS exhibits weaker direct cytotoxic efficacy against OSCC at the micromolar level. However, its advantage lies in its ability to induce vascular normalization through the MIF axis, creating a unique microenvironment remodelling effect and demonstrating a clear optimal dose window (50 mg/kg is more effective than 100 mg/kg). However, most *in vivo* oral cancer studies utilise nude mouse models, which lack T-cell infiltration validation or immunotherapy synergy in an immunocompetent setting. Furthermore, the short pharmacokinetics and poor oral solubility of the ARS monomer suggest that clinical translation will likely rely on semisynthetic derivatives (e.g., artesunate and dihydroartemisinin) or nanodelivery systems rather than the direct application of the parent metabolite.

#### 3.2.5 Cantharidin

Cantharidin (CTD) is a monoterpenoid metabolite derived from the TCM insect *Mylabris phalerata* Pallas (Coleoptera: Meloidae). Extensive studies have demonstrated its considerable potential against multiple malignancies. Reported anticancer mechanisms span inhibition of tumor-cell growth, proliferation, and metastasis; induction of apoptosis, autophagy, and pyroptosis; DNA damage with impaired repair; cell-cycle blockade; and enhanced sensitivity to radio- and chemotherapy (Naz et al., 2020). Notably, in July 2023, the U.S. Food and Drug Administration approved a 0.7% CTD topical solution for the treatment of molluscum contagiosum in adults and children ≥2 years, marking a major milestone in its clinical development (Keam, 2024). Nevertheless, CTD is associated with toxicity to vital organs, including the liver, kidney, and heart (Zhu et al., 2019; Zhang et al., 2020; He et al., 2022).

In oral cancer research (Figure 5), CTD suppresses proliferation and promotes apoptosis in Tca8113 tongue squamous cell carcinoma cells by downregulating miR-214, upregulating p53, and modulating the Bcl-2/Bax axis (Tian et al., 2015). In OSCC lines (SAS, CAL-27, SCC-4), CTD reduces JNK-dependent mitochondrial membrane potential and intensifies endoplasmic-reticulum (ER) stress signaling, thereby amplifying cytotoxicity and inducing apoptosis (Su et al., 2016). Although the mechanisms underlying CTD’s antitumor activity in oral cancer remain incompletely characterized, current evidence supports pronounced pro-apoptotic and anti-proliferative effects of CTD linked to miRNA regulation, mitochondrial dysfunction, and ER-stress pathways. Further work should focus on toxicity mitigation and targeted delivery to enable safer clinical application.

Although Triptolide and Cantharidin are both terpenoid toxin-type natural products, they exhibit significant differences in therapeutic efficacy and development positioning. In terms of antitumour potency, triptolide exhibits nanomolar efficacy (30–100 nM) in inhibiting the proliferation of OSCC, and downregulates PD-L1 under IFN-γ stimulation. This suggests not only superior potency, but also potential involvement in immune evasion mechanisms. However, its systemic toxicity (particularly hepatotoxicity) necessitates development strategies that rely on targeted delivery at low doses or approaches that detoxify derivatives. In contrast, cantharidin exhibits mostly low micromolar activity (IC_50_ = 10–30 μM) against oral cancer, demonstrating markedly weaker potency than triptolide. Its mechanism primarily centres on JNK-mediated apoptotic stress and regulation of the microRNA-p53 axis. Furthermore, its potent hepatotoxicity and nephrotoxicity as a cantharidin toxin impose significant constraints. Currently, it is more suitable as a detoxified derivative or a lead scaffold for local delivery than as a direct clinical candidate.

### 3.3 Phenolic metabolites

Phenolic metabolites comprise a diverse class of natural products characterized by one or more phenolic hydroxyl groups. They are widely distributed in TCMs and in dietary sources such as fruits, vegetables, and tea. Their anticancer activities are mediated primarily through antioxidant and anti-inflammatory actions, together with inhibition of tumor-cell proliferation and metastasis. In oral cancer research, resveratrol and curcumin are the most extensively studied representatives; the subsections below summarize their sources, principal molecular mechanisms, and key experimental evidence (Table 3).

**TABLE 3:** Mechanisms against oral cancer of phenolic metabolites, polysaccharides, alkaloids, quinones and other TCM active metabolites.

TCM active metabolites	Medicinal sources	Cell lines	*In vivo models*	Targets	Effects	References
Resveratrol 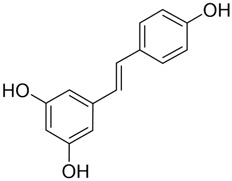	*Reynoutria japonica* Houtt. (Polygonaceae)	CAL-27	NA	↓MAGEA12, p-Akt, Cyclin D1, CDK14	↓Cell proliferation	Shang et al. (2021)
		HSC2, HSC-3, HSC-4, Ca9-22, SAS	Subcutaneous injection of Ca9-22 cells into nude mice	↑Beclin1, LC3-II; ↓SREBP1, E-FABP, p62	↓Cell viability, proliferation; ↑Lipid metabolism disorders, autophagy	Fukuda et al. (2022)
		CAL-27	NA	↓RAC1, VEGF, RGS5, CD105, ITGA5, ITGB1, CD44; ↑ZNF750, PHD2, PDGFB	↓Cell viability, proliferation, angiogenesis; ↑Cell apoptosis, migratory, invasion capacity, Vascular normalization	Xiao et al. (2021)
		HSC-3, SAS	NA	↓SNAI1; ↑E-cadherin	↓EMT; ↓Cell migratory	Min et al. (2024)
		SAS, HSC-3	Subcutaneous injection of SAS-R/Sa3-R cells into nude mice	↓uPAR, Integrinβ1, p-Src, p-FAK, p-ERK1/2	↓Cell viability, proliferation; ↑Sensitivity of cetuximab	Uzawa et al. (2019)
		CAR	NA	↓p-ERK, p-p38, MMP2−/−9	↓Cell migratory, invasion capacity	Chang et al. (2021)
Curcumin 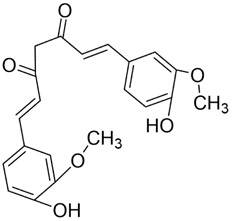	*Curcuma longa* L. (Zingiberaceae)	NA	Induction of OSCC epithelial dysplasia in Sprague-Dawley rats by DMBA	↓HO-1	↓the degree of epithelial dysplasia	Maulina et al. (2019)
		CAL-27	NA	↓Bcl2/Bax; ↑Cleaved-Caspase-3	↓Cell proliferation, migratory; ↑Cell apoptosis	Ma et al. (2020)
		SCC-25	Subcutaneous injection of SCC-25 cells into nude mice	↓BER	↓Topoisomerase activity; ↑Cell apoptosis, Olaparib Sensitivity	Molla et al. (2020)
Chlorogenic acid 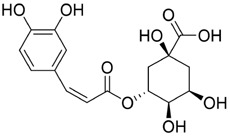	*Lonicera japonica* Thunb. (Caprifoliaceae) *Eucommia ulmoides* Oliv. (Eucommiaceae)	KB	NA	↓P53, P21	Blocking the cell cycle in the G1/S phase ↓Cell proliferation	Sharma et al. (2022)
Safflower polysaccharide	*Carthamus tinctorius* L. (Asteraceae)	HN-6	Subcutaneous injection of HN-6 cells into nude mice	↓Bcl-2, COX-2; ↑Bax, Cleaved-caspase-3	Blocking the cell cycle in the G0/G1 phase ↓Cell proliferation; ↑Cell apoptosis	Zhou et al. (2018)
Ganoderma lucidum Polysaccharides	*Ganoderma lucidum* (Curtis) P.Karst. (Ganodermataceae)	SCC-9	NA	↓TWIST, E-cadherin, Vimentin, ABCB1 et al.; ↑SNAIL, EpCAM	↓Cell proliferation, migratory, invasion capacity, EMT, CSC; ↑Chemosensitivity	de Camargo et al. (2022)
		SAS, HSC-3	NA	↓Cdc2, Cdc25C, Cytochrome C, p-EGFR, p-AKT; ↑Cyclin B1, Cleaved-caspase-3/-7/-9, Bax/Bcl-2	Blocking the cell cycle in the G2/M phase ↑Cell apoptosis, Chemosensitivity ↓Cell proliferation	Hsu et al. (2021)
Evodiamine 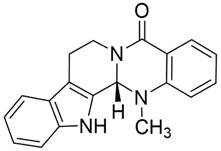	*Tetradium ruticarpum* (A.Juss.) T.G.Hartley (Rutaceae)	HSC-4	NA	↓p-AKT, Mcl-1; ↑Bax, Cleaved-Caspase-3, Cleaved-PARP	↓Cell proliferation; ↑Cell apoptosis	Sachita et al. (2015)
		CAL-27	NA	↓INF, STAT2, JAK/STAT; ↑SOCS2	↓Cell proliferation; ↑Cell apoptosis	Wu et al. (2019)
​	​	A-253, HSC-4, CAL-27, SCC-4	Subcutaneous injection of HSC-4 cells into nude mice	↓HMGB1, RAGE, NF-κB, VEGF, MMP-2	↓Cell proliferation, invasion capacity, angiogenesis	Ren et al. (2021b)
		CAL-27, SAS	Subcutaneous injection of CAL-27 cells into nude mice	↓EPRS, Glu-tRNA	↓Cell proliferation	Lin et al. (2024)
Tanshinone IIA 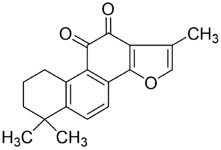	*Salvia miltiorrhiza* Bunge (Lamiaceae)	CAL-27, SCC-9, SCC-15, SCC-25	Subcutaneous injection of CAL-27/SCC-15 cells into nude mice	↓p-Akt, Ubiquitination of c-Myc, HK2; ↑GSK3β, Bax	↓Cell proliferation, glycolysis; ↑Cell apoptosis	Li et al. (2020)
		SCC-9	Subcutaneous injection of SCC-9 cells into nude mice	↓PI3K/Akt/mTOR; ↑Cleaved-caspase-3, LC3-II/-I et al.	↓Cell proliferation; ↑Cell apoptosis and autophagy	Qiu et al. (2018)
		SCC090, CAL-27	NA	↑γ-H2AX, Beclin-1, Atg5, LC3-II/-I	↑ROS, autophagy, Radiosensitivity	Ding et al. (2016)
		CAL-27, SCC-9, SCC-15, SCC-25	Subcutaneous injection of CAL-27/SCC-25 cells into nude mice	↓Aurora B, Thr232, p-H3 (Ser10); ↑Cleaved-caspase-3, Cleaved-PARP, γ-H2AX	Blocking the cell cycle in the G2/M phase ↑DNA damage, cell apoptosis	Li et al. (2021)
		CAL-27, SCC-4, SCC-25	Subcutaneous injection of CAL-27 cells into nude mice	↓p-Akt, p-Foxo3a (Ser253); ↑PUMA, Cleaved-caspase 3	↑Cell apoptosis	Han et al. (2023)
Obovatol 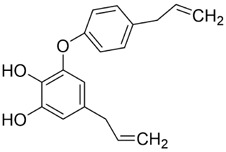	*Magnolia officinalis* Rehder and E.H.Wilson (Magnoliaceae)	SCC-9	Subcutaneous injection of SCC-9 cells into nude mice	↓CDK1, Bcl-2, p53, JAK, p-EGF, p-STAT, PDGF, VEGFR et al.; ↑ED, Caspase-3/-9, Apaf-1	Blocking the cell cycle in the G1 phase ↓Cell proliferation, migratory, invasion capacity, EMT; ↑Cell apoptosis	Duan et al. (2018)
Erianin 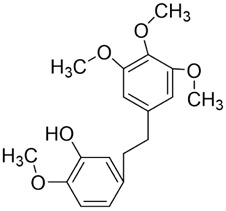	*Dendrobium nobile* Lindl. (Orchidaceae)	WSU-HN4, SCC-9, CAL-27	Subcutaneous injection of WSU-HN4 cells into nude mice	↓Cdc25c, p-Cdc2, PPT1/mTOR; ↑Cyclin B1, Cyto C, caspase-9/3, PARP cleavage, IL-1β, LC3B-II et al.	Blocking the cell cycle in the G2/M phase ↑Cell apoptosis, focal death, autophagy	Luo et al. (2021)
		SAS, CAL-27, SCC-9, OSC20	NA	↓Bcl-2/Bcl-xL, P62, P38; ↑Caspase-3/-8/-9, PARP cleavage, Bax/Bak, DR5, LC3-II/LC3-I, ERK1/2, JNK1/2	Blocking the cell cycle in the G2/M phase ↓Cell proliferation; ↑Cell apoptosis, focal death, autophagy	Chen et al. (2020d)

#### 3.3.1 Resveratrol

Resveratrol (Res) is a plant-derived antioxidant extracted from the root extract of the TCM *Reynoutria japonica* Houtt. (Polygonaceae). As a dietary supplement, resveratrol shows a favorable safety profile, and clinical studies have reported therapeutic potential across diabetes, obesity, metabolic syndrome, cancer, non-alcoholic fatty liver disease, neurological and cardiovascular disorders, inflammatory conditions, and several gynecologic diseases (Singh et al., 2019). Mild adverse events have been noted and warrant cautious clinical use (Singh et al., 2019). In cancer prevention and therapy, Res exerts pleiotropic effects via multiple molecular pathways. Principal mechanisms include induction of cell-cycle arrest, inhibition of proliferation and metastasis, activation of apoptosis and autophagy, modulation of gut microbiota and immune responses, and attenuation of inflammatory signaling (Wu S. X. et al., 2023).

In recent years, substantial evidence has accumulated regarding the role of Res in the prevention and treatment of oral cancer (Figure 5). Inhibition of cell proliferation and cell-cycle progression: Melanoma-associated antigen A12 (MAGEA12) is implicated in head and neck squamous cell carcinoma (HNSCC) and correlates with poor survival (Habib et al., 2022). Res downregulates MAGEA12, inhibits Akt phosphorylation, and reduces Cyclin D1 and CDK14, thereby suppressing CAL-27 proliferation (Shang et al., 2021). Induction of apoptosis and autophagy: Res selectively induces autophagy in Ca9-22 cells by inhibiting sterol regulatory element-binding protein 1 (SREBP1) survival signaling (Fukuda et al., 2022), and promotes apoptosis in CAL-27 cells (Xiao et al., 2021). Suppression of angiogenesis and modulation of the tumor microenvironment: A conditioned medium enriched in M1-like macrophages—generated by co-culturing H357 cells with THP-1 acute monocytic leukemia cells—enhance cancer stemness, metastasis, and angiogenesis in primary oral-cancer cells; treatment with resveratrol nanoparticles normalizes vascular formation and improves the M1-associated inflammatory microenvironment, reducing metastasis and angiogenic marker expression (Pradhan et al., 2021). Regulation of inflammation and microbiota: *Fusobacterium* nucleatum promotes tumor progression via proliferation, chemoresistance, immune remodeling, immune-checkpoint activation, and metastasis (Alon-Maimon et al., 2022). In HSC-3 and SAS cells, Res counteracts *Fusobacterium* licheniformis–induced EMT by downregulating SNAI1 and upregulating E-cadherin (Min et al., 2024). Reversal of drug resistance: The urokinase-type plasminogen activator receptor (uPAR) is frequently overexpressed and linked to therapy resistance (Zhai et al., 2022). Res enhances the sensitivity of SAS and HSC-3 cells to cetuximab by suppressing uPAR and overcomes cisplatin resistance via inhibition of ERK/p38 signaling and MMP-9/-2, thereby reducing migration and invasion (Uzawa et al., 2019; Chang et al., 2021). Collectively, Res displays multifaceted anticancer activity in oral cancer by modulating pathways governing proliferation, apoptosis, autophagy, angiogenesis, inflammation, and drug resistance. The above studies indicate that resveratrol exhibits anti-tumour effects within the 10–100 μM range for the treatment of oral cancer. Specifically, it significantly inhibits Ca9-22 cells at around 50 μM, suppresses invasion in drug-resistant models within the 25–75 μM range and can overcome Cetuximab resistance. However, its relatively high effective concentration and unclear *in vivo* exposure window limit its direct translation as a monotherapy. Advances in resveratrol-based nanomedicines have improved bioavailability and tumor targeting, supporting translational development.

#### 3.3.2 Curcumin

Curcumin (Cur) is a bioactive polyphenolic metabolite sourced from *Curcuma longa* L. (Zingiberaceae), a traditional Chinese medicinal botanical drug. Owing to its broad pharmacological profile, Cur has become a major focus in natural-product and drug-discovery research. Extensive evidence indicates therapeutic potential across malignancies, including lung, prostate, breast cancer and HNSCC (Hao M. et al., 2023). In oncology, Cur exerts pleiotropic effects via multiple pathways; principal mechanisms include inhibition of tumor-cell proliferation and cell-cycle progression, regulation of programmed cell death, suppression of EMT, and enhancement of cell–cell adhesion (Wang W. et al., 2023).

Both *in vitro* and *in vivo* studies have confirmed the anticancer potential of Cur in oral cancer (Figure 5). Cur suppresses the proliferation of oral cancer cells by interfering with DNA synthesis and inducing cell-cycle arrest (Ravera et al., 2024). Cur exhibits dose-dependent inhibition of proliferation in CAL-27 cell, significantly suppressing migration within the 50–100 μM range, causing S-phase arrest and reducing colony-forming capacity (Ma et al., 2020). Similarly, the curcumin derivative PAC inhibits growth by enforcing cell-cycle blockade and activating autophagy in Ca9-22 cells (Semlali et al., 2021). In addition to proliferation inhibition, Cur induces apoptosis in CAL-27 cells by activating cleaved caspase-3 and decreasing the Bcl-2/Bax ratio (Ma et al., 2020), and selectively reduces the viability of TSCC-associated fibroblasts without affecting normal epithelial cells (Hussein and Khaphi, 2023). The derivative HO-3867 triggers sub-G1 arrest in SCC-9 and HSC-3, enhances phosphorylation of ERK1/2, JNK1/2, and p38, and promotes apoptosis (Chen et al., 2022). Moreover, in SAS cells, Cur nanoparticles combined with gefitinib markedly enhance apoptosis through caspase and mitochondria-dependent pathways, with superior efficacy in xenograft models (Lai et al., 2019). In Ca9-22, PAC induces both apoptosis and autophagy, accompanied by loss of mitochondrial membrane potential and altered oxidative-stress levels (Semlali et al., 2021). Beyond these effects, Cur attenuates EMT and metastasis, reducing invasive and migratory potential (Ma et al., 2020). In parallel, in a DMBA-induced rat oral-cancer model, Cur lessens epithelial dysplasia and increases heme oxygenase-1 (HO-1) expression, delaying OSCC progression (Maulina et al., 2019). *In vitro*, Cur further suppresses oxidative stress by inhibiting the NF-κB and Wnt signaling pathways (Semlali et al., 2021). In addition, the IC_50_ of Cur as a single agent ranges from approximately 15–50 μM. However, co-treatment with the PARP inhibitor olaparib at concentrations of just 10 μM Cur and 2 nM olaparib exacerbates DNA damage, inhibits topoisomerase activity, and augments apoptosis *in vitro* (Molla et al., 2020). Likewise, Cur nanoparticles plus gefitinib yield stronger cytotoxic and pro-apoptotic effects than either agent alone, consistent across *in vitro* and *in vivo* settings (Lai et al., 2019). Collectively, Cur shows potent, multitarget anticancer activity in oral cancer by modulating proliferation, apoptosis, autophagy, EMT, oxidative stress, and therapeutic resistance. However, its relatively high effective concentration when used as a monotherapy, as well as its unclear bioavailability and exposure window, make it a better candidate for use in combination with other therapies to improve sensitisation or optimise delivery.

#### 3.3.3 Chlorogenic acid

Chlorogenic acid (CGA) is a natural polyphenolic metabolite present in several traditional Chinese medicinal botanical drugs, including *Lonicera japonica* Thunb. (Caprifoliaceae) and *Eucommia ulmoides* Oliv (Eucommiaceae). It exhibits diverse biological activities, including antioxidant and antibacterial effects, regulation of glucose and lipid metabolism, and protection of the cardiovascular, cerebrovascular, and nervous systems (Naveed et al., 2018). Anticancer effects include induction of apoptosis; inhibition of tumor-cell proliferation, migration, and invasion; suppression of angiogenesis; and enhancement of chemosensitivity (Gupta et al., 2022). Interest in CGA for oral-cancer prevention and therapy has grown in recent years (Figure 5). Integrated computational analyses implicate MAPK-ERK/JNK signaling and key molecular targets such as MMP-9, SRC, and ERBB2. Through these interactions, CGA modulates proliferation, migration, adhesion, and apoptosis, suggesting potential to hinder OSCC progression (Feng et al., 2024). Subsequent *in vitro* studies further confirmed that CGA effectively suppresses the proliferation of KB cells by downregulating p53 and p21 expression, leading to G1/S-phase arrest. Notably, this effect reflects growth inhibition rather than direct cytotoxicity (Sharma et al., 2022). In summary, CGA is a naturally abundant, multitarget candidate against oral cancer.

Overall, the evidence for the antitumour properties of chlorogenic acid in oral cancer remains relatively weak, exhibiting the characteristic profile of a “low-toxicity, low-efficacy” polyphenol. Existing studies indicate an IC_50_ of approximately 1.8 mM in KB cells, meaning millimolar concentrations are required to achieve 50% inhibition. Furthermore, the lack of evidence for apoptosis or DNA fragmentation suggests that its primary mechanism is the inhibition of non-specific proliferation rather than the induction of therapeutic cytotoxicity. By contrast, polyphenols such as resveratrol and curcumin typically exert effects at concentrations of 10–100 μM, which highlights a significant efficacy gap for CGA. The clinical feasibility of CGA in terms of exposure and translational potential remains questionable. Moreover, the existing data are limited to a single cell line and lack *in vivo* validation, pharmacokinetic bridging and comparisons with standard controls. This makes CGA more suitable as a dietary chemopreventive agent or co-regulatory molecule. Future research should prioritise enhancing delivery, developing combination strategies, and establishing *in vivo* exposure-response relationships.

### 3.4 Polysaccharides

In TCMs, botanical drug-derived polysaccharides have attracted growing interest in oncology. Owing to their robust immunomodulatory capacity, these polymers exhibit broad pharmacological activities for therapeutic development and clinical translation of oral cancer (Table 3).

#### 3.4.1 Safflower polysaccharide

Safflower polysaccharide (SPS) refers to water-soluble heteropolysaccharides extracted from *Carthamus tinctorius* L. (Asteraceae) Modern pharmacology indicates significant immunomodulatory, antioxidant, anticancer, neuroprotective, and osteoprotective (Wu et al., 2021). In recent years, SPS has attracted increasing interest in oncology owing to broad tumor-suppressive potential across multiple solid-tumor models. SPS inhibits inflammation-related cytokines and cancer-cell proliferation in colorectal cancer (Wang Q. et al., 2021); induces apoptosis, causes G2/M phase arrest, and enhances immune regulation in lung cancer models (Li et al., 2016); activates cytotoxic T lymphocyte (CTL) and natural killer (NK) cell functions (Shi et al., 2010), and suppresses breast-cancer proliferation and metastasis (Luo et al., 2015).

**FIGURE 6:**
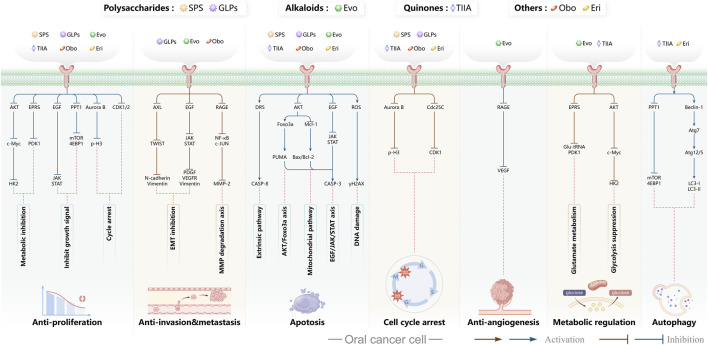
Mechanisms of action for other metabolites extracted from traditional Chinese medicine against oral cancer. These include polysaccharides—Safflower polysaccharide (SPS), Ganoderma lucidum Polysaccharides (GLPs)—as well as alkaloids—Evodiamine (Evo), quinones—Tanshinone IIA (TIIA), and metabolites such as Obovatol (Obo) and Erianin (Eri). Their anti-oral cancer effects primarily manifest through mechanisms including antiproliferative activity, inhibition of migration and invasion, induction of apoptosis, cell cycle arrest, anti-angiogenesis, metabolic regulation, and induction of autophagy.

Research on oral cancer has shown that SPS significantly inhibits proliferation of HN-6 human TSCC cells *in vitro* and *in vivo* (Figure 6). Mechanistically, it downregulates Bcl-2 and COX-2 while upregulating Bax and cleaved caspase-3, inducing apoptosis and G0/G1-phase arrest (Zhou et al., 2018). Current oral-cancer studies are limited. *In vitro*, SPS requires concentrations ranging from 20 to 640 μg/mL to inhibit the proliferation of HN-6 cells in a squamous cell carcinoma of the tongue model. *In vivo*, the daily administration of 40 mg/kg in a xenograft model reduces tumour volume without causing significant changes in body weight. However, compared to most small-molecule natural products that exhibit efficacy at the μM level, SPS requires significantly higher doses, which suggests that it probably relies on non-specific or immune/inflammatory modulation rather than potent cytotoxic activity. Furthermore, this study pertains to a plant extract rather than a pure metabolite. While it details a hot water extraction, ethanol precipitation and Sevage deproteinisation process, and identifies glucose (97%) as the primary metabolite, the lack of molecular weight distribution, structural characterisation and pharmacokinetic data makes it more suitable as a candidate for adjunctive immunomodulation or combination therapy.

#### 3.4.2 Ganoderma lucidum polysaccharides

Ganoderma lucidum Polysaccharides (GLPs) are principal bioactive metabolites of *Ganoderma lucidum* (Curtis) P. Karst. (Ganodermataceae), a renowned traditional Chinese medicinal fungus. They exhibit immunomodulatory, anticancer, antioxidant, anti-aging, hypoglycemic, cardioprotective, and hepatoprotective activities (Luo et al., 2022). Anticancer actions are mediated predominantly via immune regulation and also involve direct interference with tumor-cell adhesion, invasion, and metastasis; induction of apoptosis and cell-cycle arrest; and suppression of angiogenesis (Gao and Homayoonfal, 2023).

GLPs markedly inhibit the growth, migration, and invasion of SCC-9 cells (Figure 6). In addition, GLPs restrict malignant proliferation by eliciting cellular senescence. Mechanistically, they suppress EMT and downregulate cancer stem cell (CSC) -associated markers, partially reversing malignant phenotypes. GLPs also reduce the expression of ATP-binding cassette (ABC) transporters such as ABCB1, suggesting potential to mitigate multidrug resistance and enhance chemosensitivity (de Camargo et al., 2022). WSG, a defined GLP subtype, shows potent anti-oral-cancer activity: it suppresses proliferation of SAS and HSC-3 cells by inhibiting EGFR/AKT signaling, induces G2/M-phase arrest, and promotes mitochondrial-dependent apoptosis via modulation of the Bax/Bcl-2 ratio and activation of caspases-3/7/9. Notably, WSG displays synergy with cisplatin and simultaneously protects normal human gingival (SG) cells (Hsu et al., 2021). In summary, GLPs act through multitarget and multipathway mechanisms in oral cancer, constraining tumor progression and enhancing the efficacy of radio- and chemotherapy.

In summary, GLPS/WSG exhibits certain anti-tumour potential in oral cancer, but its overall therapeutic window remains relatively narrow. WSG demonstrated IC_50_ values of 107 μg/mL and 232 μg/mL in SAS and HSC3 tongue cancer cells, respectively. These values indicate that exposure levels in the hundreds of micrograms per millilitre are required to inhibit proliferation and induce apoptosis. This potency level is comparable to that of SPS, which requires 20–640 μg/mL to exhibit inhibitory effects in HN-6 cells. This suggests that polysaccharide metabolites generally lack the direct cytotoxic activity at the μM level observed in small molecules such as resveratrol or curcumin. Conversely, polysaccharides appear to be more effective in modulating invasion phenotypes, CSC/EMT and microenvironment-related pathways. However, most such studies involve polysaccharides from extracts rather than purified monomers. Despite some characterisation, the lack of structure–activity relationships, *in vivo* pharmacokinetic exposure data and batch standardisation limits their translational potential, rendering them more suitable as immune adjuvants or metabolites of combination therapies.

### 3.5 Alkaloids

Alkaloids are nitrogen-containing natural metabolites widely utilized in TCMs for their diverse pharmacological activities, including antibacterial, anti-inflammatory, and anticancer effects (Table 3).

Evodiamine (Evo) is an active alkaloid isolated from the fruit of *Tetradium ruticarpum* (A.Juss.) T.G.Hartley (Rutaceae). It exhibits anticancer, antimicrobial, cardioprotective, hepatoprotective, renoprotective, metabolic-regulatory, and neuroprotective activities. However, its pharmacokinetics warrant attention, as prolonged accumulation or rapid administration may cause cardiac and hepatic toxicity (Sun et al., 2020). Evo shows potent antitumor activity across lung (Jiang et al., 2020), bladder (Hu C. Y. et al., 2023), colorectal (Zhu L. Q. et al., 2021), liver (Zhao et al., 2020), oral (Wu et al., 2019), pancreatic (Hong et al., 2020), thyroid (Chien et al., 2021), and prostate cancers (Hwang et al., 2020). Its anticancer mechanisms primarily involve inhibition of proliferation, migration, and invasion (Wang et al., 2019); induction of apoptosis (Hong et al., 2020; Hwang et al., 2020; Chien et al., 2021), autophagy (Wang et al., 2019); modulation of angiogenesis (Hu C. Y. et al., 2023; Yu et al., 2023), ferroptosis (Hwang et al., 2020) and the tumor immune microenvironment (Jiang et al., 2020). Additionally, evodiamine may influence tumorigenesis by regulating gut microbiota composition (Zhu L. Q. et al., 2021).

In oral cancer (Figure 6), Evo (0.2–20 μM) suppresses proliferation of HSC-4 cells and induces apoptosis by downregulating Mcl-1 via AKT pathway inhibition (Sachita et al., 2015). Transcriptomic/bioinformatic analyses indicate that Evo suppresses interferon signaling in CAL-27 cells, thereby inhibiting growth and cell-cycle progression while promoting apoptosis (Wu et al., 2019). Moreover, in multiple OSCC lines, the receptor for advanced glycation end products (RAGE) and its ligand HMGB1—molecules associated with migration, invasion, and poor prognosis—are highly expressed (Sims et al., 2010). Evo downregulates RAGE, HMGB1, VEGF, and MMP-2, thereby inhibiting proliferation, invasion, and angiogenesis in A-253 and TSCC lines (HSC-4, CAL-27, SCC-4) (Ren L. et al., 2021). Furthermore, glutamyl-prolyl-tRNA synthetase (EPRS) is overexpressed in OSCC; both *in vivo* and *in vitro* studies demonstrate that Evo downregulates EPRS and inhibits proliferation, potentially through transcriptional regulation, although the precise mechanism remains to be elucidated (Lin et al., 2024). In summary, evodiamine exhibits clear *in vitro* activity against OSCC (Cal27/SAS IC50: 9.7–14.4 μM) and inhibits proliferation and invasion by targeting the AKT-Mcl-1 apoptosis axis, as well as the HMGB1/RAGE and EPRS metabolic pathways (Lin et al., 2024). However, existing *in vivo* evidence is primarily limited to nude mouse xenografts with a broad dose range of 3–20 mg/kg (Ren L. et al., 2021; Lin et al., 2024). High doses exhibit signs of weight loss, and the exposure-safety window and primary target remain unclear. Therefore, rather than positioning Evo as a treatment molecule ready for direct advancement, it should be regarded as a natural product skeleton offering mechanistic clues, but requiring continuous optimization through systematic pharmacokinetic studies, safety evaluations, and model validation.

### 3.6 Quinones

Tanshinone IIA (TIIA) is the principal lipophilic bioactive metabolite extracted from the roots of *Salvia miltiorrhiza* Bunge (Lamiaceae). It exhibits a broad pharmacological profile (cardiovascular and cerebrovascular protection, anti-diabetic and anti-obesity effects, neuroprotection, and anticancer activity) (Guo et al., 2020; Ansari et al., 2021). Anticancer mechanisms of TIIA (Table 3) are multifaceted and include cell-cycle regulation, inhibition of metastasis and invasion, induction of apoptosis and suppression of angiogenesis (Fang et al., 2020).

Enhanced glycolysis is a metabolic hallmark that fuels rapid proliferation in OSCC and other malignancies (Gatenby and Gillies, 2004) (Figure 6). This reprogramming also promotes immune evasion by upregulating PD-L1 via hexokinase-2 (HK2) activation (Guo et al., 2022). Among OSCC lines, CAL-27 displays the highest glycolytic activity with pronounced HK2 overexpression. TIIA suppresses glycolysis in CAL-27, SCC-9, SCC-15, and SCC-25 cells and reduces HK2 expression by blocking the AKT–c-Myc axis, thereby inhibiting proliferation and survival (Li et al., 2020). Beyond this, TIIA triggers apoptosis and autophagy. In SCC-9 cells, it concurrently inhibits PI3K/AKT/mTOR and activates the Beclin-1/Atg7 cascade, inducing apoptosis and autophagy (Qiu et al., 2018). Notably, in CAL-27, SCC-4, and SCC-25 cells, TIIA primarily acts via the AKT–FOXO3a pathway to upregulate PUMA and promote apoptosis independently of autophagy (Han et al., 2023). In addition, in SCC-9, SCC-15, SCC-25, and CAL-27 cells, TIIA inhibits Aurora B kinase activity, inducing G2/M-phase arrest and enhancing apoptosis (Li et al., 2021). Importantly, TIIA exhibits selective cytotoxicity toward tumor cells while sparing normal tissues and retains activity in radioresistant OSCC. It enhances radiosensitivity by increasing ROS and elevating autophagy (Ding et al., 2016). In summary, TIIA exerts multitarget anticancer effects in OSCC—remodeling glycolytic metabolism, inducing apoptosis and/or autophagy, inhibiting key cell-cycle regulators, and enhancing radiosensitivity. Further studies across multiple OSCC lines and *in vivo* models are warranted to validate mechanisms, define translational potential, and optimize combination strategies for efficacy and safety. The above evidence on oral cancer suggests that tanshinone IIA exhibits stable anti-tumor properties at low micromolar concentrations *in vitro*. In CAL-27, SCC4, and SCC-25 cells, it demonstrates dose-dependent inhibition at concentrations of 0–5 μM. At 5 μM, it nearly blocks clonogenic formation. The IC_50_ in SCC-9 cells is approximately 17.54 μM. *In vivo* studies using most xenograft models demonstrated a 50%–70% reduction in tumor weight following intraperitoneal injections of 10 or 30 mg/kg, with no significant changes in body weight, blood test results, or major organ pathology. These results suggest a feasible therapeutic window at the mouse level. However, the current evidence remains largely confined to the stage of “effective against xenografts + multiple concurrent mechanisms.” Further validation of exposure-response relationships, *in situ* microenvironments, and immune contexts is required to determine its true translational value.

### 3.7 Others

Obovatol (Obo) (Table 3) is a lignan derived from *Magnolia officinalis* Rehder and E.H.Wilson (Magnoliaceae), a traditional Chinese medicinal botanical drug. It exhibits diverse pharmacological activities with minimal toxicity to normal cells. Reported anticancer mechanisms include inhibition of tumor-cell proliferation, suppression of migration and invasion, induction of apoptosis, and inhibition of angiogenesis (Lee et al., 2011). In oral cancer (Figure 6), Obo shows marked cytotoxicity against SCC-9 TSCC cells. Mechanistically, Obo reduces proliferation and invasiveness of SCC-9 cells by downregulating epidermal growth factor and key metabolites of the JAK/STAT pathway, and it modulates apoptosis-associated genes (caspase-3, caspase-9, and Apaf-1) to promote apoptosis (Duan et al., 2018). As a low-toxicity natural product with broad bioactivity, Obo merits further investigation as a potential therapeutic candidate in oral cancer.

Erianin (Eri) (Table 3) is a naturally occurring bibenzyl metabolite primarily isolated from *Dendrobium nobile* Lindl. (Orchidaceae) (Chen P. et al., 2020). It displays potent antitumor activity and exerts effects via multiple mechanisms, including inhibition of proliferation, induction of apoptosis and autophagy, suppression of angiogenesis and inhibition of metastasis (Wei et al., 2024). In oral cancer (Figure 6), Eri suppresses proliferation of OSCC cell lines such as WSU-HN4, SCC-9, and CAL-27, and inhibits tumor growth *in vivo*. Mechanistically, Eri inhibits the PPT1–mTOR axis, induces multimodal cell death and blocks protective autophagy (Luo et al., 2021). In addition, Eri regulates MAPK signaling, induces G2/M-phase arrest, and activates apoptosis and autophagy in SCC-9, CAL-27, and OSC20 cells, thereby enhancing anticancer activity (Chen Y. T. et al., 2020). While evidence supports regulating oncogenic signaling and programmed cell death in multiple ways, further pharmacokinetic studies, *in vivo* validation, and well-designed preclinical trials are needed to enable clinical translation.

Overall, Erianin demonstrates a more favorable pharmacodynamic profile and mechanism of action in OSCC. Early observations of its cytotoxicity at the micromolar level (25–100 μM), followed by subsequent activity at the nanomolar level (50–100 nM), coupled with its targeting of the PPT1/mTOR axis, induction of apoptosis-autophagy blockade, and pyroptosis, suggest that it may be a promising lead metabolite with development potential. In contrast, although obovatol demonstrated tumor suppression in xenograft models with short-course administration at 5 mg/kg, its *in vitro* testing relied solely on a single, high concentration, and lacked defined PK/toxicity windows. This positions Obovatol closer to a mechanism-validating natural molecule than a mature translational candidate. Future development strategies for Erianin should focus on optimizing delivery and reducing exposure, while foundational evidence on dose-response and safety profiles must first be established for Obovatol before advancing to clinical development.

## 4 Limitations and future research directions

### 4.1 Limitations of preclinical evidence and model systems

Although natural bioactive metabolites have demonstrated multi-target regulation and promising anti-tumor potential in preclinical oral cancer studies, existing evidence is primarily limited to *in vitro* cell experiments and mouse subcutaneous xenograft models (Acharya et al., 2024), which limits the ability to extrapolate research conclusions to clinical settings. Many conclusions are drawn from simplified *in vitro* assays that often lack standardized IC_50_ reporting, rigorous positive controls, and clinically realistic exposure ranges. While commonly used OSCC cell lines (e.g., CAL-27, SCC-9, HSC-3, and SAS) facilitate mechanism validation, they inadequately reflect tumor heterogeneity, the local oral microenvironment, immune infiltration, and metastatic processes (Piwowarczyk et al., 2022). Conclusions about “immune microenvironment regulation” derived from immunodeficient animal models need to be validated using immunocompetent and *in situ* models (Kono et al., 2022). Therefore, future studies should prioritize oral *in situ* tumor models, patient-derived organoids/PDX systems, and validation frameworks under combined chemoradiotherapy settings to enhance translational relevance.

### 4.2 PAINS-related artifacts and overestimation of pharmacological relevance

A key methodological concern is that many well-studied flavonoids and polyphenols are classified as PAINS (Magalhães et al., 2021). These metabolites can produce false *in vitro* activity through non-specific mechanisms, such as redox cycling, aggregation, fluorescence interference, or promiscuous binding (Bolz et al., 2021). Therefore, pathway-level effects observed in cell assays may not reflect true target-specific pharmacology. Quercetin is a typical example. Despite frequent reports of multi-pathway inhibition in OSCC models, its well-recognized assay interference liabilities necessitate cautious interpretation of many high-concentration *in vitro* claims, as they may overestimate therapeutic relevance.

### 4.3 Pharmacokinetics, safety, and delivery challenges

The core bottleneck in the translation of natural products lies in the insufficient evidence of PK safety. Many metabolites exhibit clear *in vitro* activity, yet encounter barriers during translation due to poor solubility, low absorption rates, rapid *in vivo* clearance, and inadequate tissue exposure (Ren B. et al., 2021). This results in “unattainable effective concentrations”. Furthermore, certain natural metabolites exhibit potential organ toxicity or narrow therapeutic windows (Rani et al., 2024), necessitating a systematic evaluation of the maximum tolerated dose, repeated-dose toxicity, drug interactions, and long-term safety in order to establish predictable exposure-effect relationships. Formulations and delivery systems (e.g., nanocarriers) can significantly enhance bioavailability, targeting, and toxicity reduction (Hu et al., 2025). However, they may introduce new challenges, such as carrier toxicity, uncontrolled *in vivo* distribution, and batch-to-batch variability. Therefore, their evaluation should focus on “enhancing tumor exposure while reducing systemic toxicity” rather than merely increasing *in vitro* uptake or achieving conceptual innovation.

### 4.4 Clinical translation pathways and future research priorities

Clinical research data on the treatment of oral cancer with natural bioactive metabolites was retrieved from the Clinical Research Network website (https://clinicaltrials.gov/) (Table 4). A large number of high-quality natural bioactive metabolites have been used in studies of head and neck tumors, including oral cancer. These trials indicate emerging clinical interest, but efficacy remains unproven and requires rigorous validation. These findings suggest that future research on natural products for oral cancer should advance along the “manufacturability-PK/toxicity predictability-combination therapy-oriented clinical validation” pathway, rather than remaining confined to pathway summaries at the mechanism level.

**TABLE 4:** Clinical trial of oral cancer with natural bioactive metabolites.

Project title	Compound	Status	Registration No.
Neoadjuvant Tislelizumab in Combination With Dasatinib and Quercetin in Resectable HNSCC (COIS-01)	Quercetin	Phase 2	NCT05724329
Therapeutic Efficacy of Quercetin Versus Its Encapsulated Nanoparticle on Tongue Squamous Cell Carcinoma Cell Line	Quercetin	Phase 2	NCT05456022
Phase I Chemoprevention Trial With Green Tea Polyphenon E and Erlotinib in Patients With Premalignant Lesions of the Head and Neck	EGCG	Phase 1	NCT01116336
Safety and Efficacy Trial of TV5M01 for Radiation-Induced Mucositis in Head and Neck Cancer Patients. (TEMOR)	EGCG	Phase 1 Phase 2	NCT06875791
Soy Isoflavone in Combination With Radiation Therapy and Cisplatin in SCC of the Head and Neck	Genistein	Phase 1	NCT02075112
The Effect of Curcumin for Treatment of Cancer Anorexia-Cachexia Syndrome in Patients With Stage III-IV of Head and Neck Cancer (CurChexia)	Curcumin	Phase 2	NCT04208334
Curcumin Biomarker Trial in Head and Neck Cancer	Curcumin	Early Phase 1	NCT01160302

Based on the current state of research, the following priority directions for future studies can be summarized: First, transitioning from descriptive associations to causal target validation using orthogonal assays. Second, adopting PAINS-aware designs that include counter-screens and PK-supported concentration windows. Third, validating efficacy in advanced models, such as orthotopic tumors, patient-derived organoids, and immunocompetent systems. Fourth, treating pharmacokinetics and toxicity as hard thresholds. Fifth, prioritizing delivery optimization, structural modification, and rational combination strategies to enable well-designed clinical trials. Prioritize delivery optimization, structural modification, and rational combination strategies to enable well-designed clinical trials. In summary, addressing PAINS-related artifacts and enhancing mechanistic and translational standards are essential steps in advancing natural metabolites into clinically actionable therapies for oral cancer.

## 5 Conclusion

**FIGURE 7:**
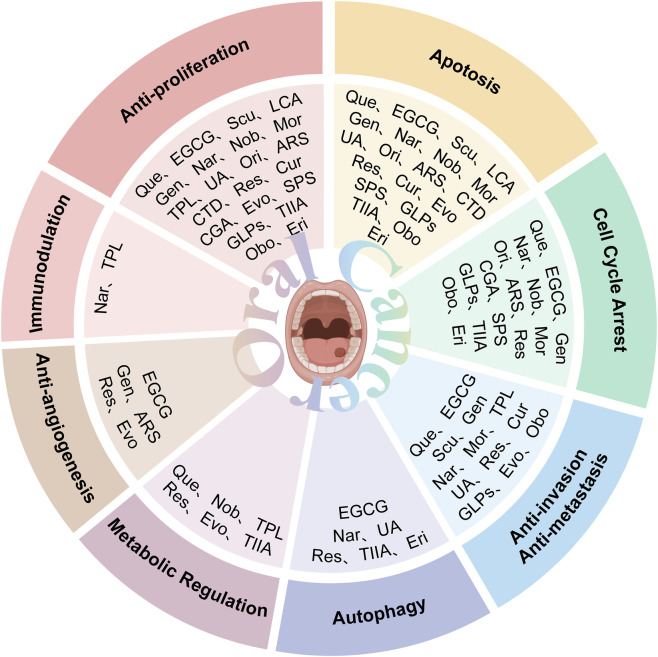
Key mechanisms of TCM active metabolites against oral cancer.

In summary, this review selects and presents natural product metabolites, particularly those from TCM, including flavonoids, terpenoids, phenolic metabolites, alkaloids, polysaccharides, and quinones. The selection is based on well-established, reproducible evidence supporting their role in oral cancer research. These metabolites have clear origins, strong representativeness, and demonstrate anti-oral cancer activity at different levels of pharmacological effects *in vitro* and in some *in vivo* models. Reported effects include growth inhibition, apoptosis induction, cell-cycle arrest, suppression of invasion and metastasis, regulation of autophagy, metabolic remodeling, anti-angiogenic activity, and modulation of the tumor immune microenvironment (Figure 7). Notably, structurally distinct metabolite classes may act synergistically within shared regulatory networks, offering a potential advantage over single-target therapies.

Despite this promise, the majority of studies remain in the preclinical stage, with limited validation of key targets and core regulatory pathways in patients. Translational gaps emerge due to suboptimal pharmaceutical properties, including poor solubility and absorption, rapid *in vivo* clearance, and potential toxicities. For instance, the oral pharmacokinetics of quercetin, resveratrol, and curcumin vary considerably, impeding the extrapolation of preclinical efficacy to clinical benefit. In a similar vein, the dose-dependent toxicities of triptolide and cantharidin constrain their clinical application. Efforts have been made to address these issues through engineering, structure optimization, derivative synthesis, and combinations, to improve safety and efficacy (Table 5).

**TABLE 5:** Optimized formulations, advantages and effects of TCM active metabolites.

TCM active metabolites	Optimized formulations	Advantages	Effects	References
Quercetin	Nanostructured lipid carriers (NLCs) with piperine	Enhanced physicochemical stability Superior bioavailability and controlled drug release Promoted cellular internalization and targeted distribution Synergistic modulation of multiple anticancer pathways	It induced apoptosis by reducing the mitochondrial membrane potential, releasing pro-apoptotic factors, and activating the caspase cascade	Chaudhari et al. (2021)
Genistein	Genistein loaded lactalbumin nanoparticles (GLNPs)	Favorable drug delivery properties Excellent biocompatibility and *in vivo* safety Synergistic Enhancement of Antitumor Potency and Selectivity	It selectively induced apoptosis and reverse epigenetic suppression in OSCC by targeting the 3 PK-EZH2 signaling axis	Dev et al. (2021)
Ursolic acid	Ursolic acid–loaded chitosan nanoparticles (UACNP)	Favorable efficacy, low toxicity, and excellent bioavailability Synergistic suppression of tumorigenesis Superior prophylactic over therapeutic efficacy	It effectively suppressed DMBA-induced oral cancer by inhibiting lipid peroxidation and modulating detoxification pathways	Karthik et al. (2023)
Resveratrol	Resveratrol nanoparticle (Res–Nano)	Improved bioavailability, stability, safety, and efficacy Enhanced targeting and cellular internalization Multi-dimensional suppression of tumor progression	It targets M1 macrophages, inhibiting cytokine release and NF-κB/AKT/MAPK signaling to suppress cancer stemness, metastasis, and angiogenesis	Pradhan et al. (2021)
Curcumin	The novel bioactive curcumin analog: 3,5-Bis (4-hydroxy-3-methoxybenzylidene)-N-methyl-4-piperidone (PAC)	Favorable safety profile over conventional therapeutics A synergistic, multi-targeted mechanism of action Selective tumor cell cytotoxicity	It inhibited the proliferation, survival, and metastasis of oral cancer cells by modulating the activity of ERK1/2, p38/JNK, NF-κB, and Wnt signaling pathways	Semlali et al. (2021)
	The novel bioactive curcumin analog: HO-3867	High potency and low toxicity A synergistic, multi-targeted mechanism of action Precise targeting of key oncogenic pathways	It induced apoptosis in oral cancer cells by activating the MAPKs signaling pathway, evidenced through increased phosphorylation of ERK1/2, JNK, and p38	Chen et al. (2022)
	γ-PGA-Gef/Cur NPs	High drug encapsulation efficiency and stability Enhanced Targeting, cellular internalization, and Minimal Toxicity Synergistically induced apoptosis Excellent aqueous solubility and oral bioavailability	It induced apoptosis through both caspase-dependent and mitochondrial pathways, leading to significant inhibition of oral cancer growth	Lai et al. (2019)

In the future, natural bioactive metabolites are expected to play a significant role in the development of new therapeutic agents for oral cancer. The following priorities have been identified: (i) mechanistic elucidation using integrative strategies, (ii) refinement of delivery technologies to enhance exposure and selectivity, (iii) rational combination designs guided by pharmacodynamics and resistance biology, and (iv) rigorously designed clinical trials with biomarker endpoints to bridge preclinical findings to patient benefit. With meticulous coordination among these domains, TCM-derived agents are strategically poised to surmount prevailing translational impediments and, in the end, furnish more efficacious and safer alternatives for patients afflicted with oral cancer.

## Appendix

### Classification of frequently used cell lines

Human tongue squamous cell carcinoma: YD10B, OSC-20, SAS, SCC-4, SCC-9, SCC-15, SCC-25, Tca8113, CAL-27, TSCCA, TSC-15, HSC-3, HSC-4, HSC-6, KBV200, KB, WSU-HN6, HN-6, HN31, H357.

Human gingival squamous cell carcinoma: YD38, Ca9-22.

Human laryngeal squamous cell carcinoma: SqCC/Y1 (Y1).

Laryngeal/Hypopharyngeal squamous cell carcinoma: Tu212, JHU-011, FaDu.

Tongue/floor of mouth carcinoma: UM1, UM2.

Salivary gland carcinoma: A-253.

Mouse oral squamous cell carcinoma: NR-S1, SCC VII (SCC7).

Other head and neck squamous cell carcinoma: MDA686TU, HN22, SCC-2095.

Oral leukoplakia: MSK-Leuk1.

Normal human oral epithelial: HOECs.
